# Alteration of endosomal trafficking is associated with early-onset parkinsonism caused by *SYNJ1* mutations

**DOI:** 10.1038/s41419-018-0410-7

**Published:** 2018-03-07

**Authors:** Dominga Fasano, Silvia Parisi, Giovanna Maria Pierantoni, Anna De Rosa, Marina Picillo, Giuseppina Amodio, Maria Teresa Pellecchia, Paolo Barone, Ornella Moltedo, Vincenzo Bonifati, Giuseppe De Michele, Lucio Nitsch, Paolo Remondelli, Chiara Criscuolo, Simona Paladino

**Affiliations:** 1 Department of Molecular Medicine and Medical Biotechnology, University of Naples Federico II, Naples, Italy; 2 Department of Neuroscience, Reproductive, and Odontostomatological Sciences, University of Naples Federico II, Naples, Italy; 3 Center for Neurodegenerative Diseases (CEMAND), Department of Medicine, Surgery and Dentistry, Neuroscience Section, University of Salerno, Salerno, Italy; 4 Department of Medicine, Surgery and Dentistry “Scuola Medica Salernitana”, University of Salerno, Salerno, Italy; 5 Department of Pharmacy, University of Salerno, Salerno, Italy; 6 Department of Clinical Genetics, Erasmus MC, Rotterdam, The Netherlands; 7 CEINGE Biotecnologie Avanzate scarl, Naples, Italy

## Abstract

Recently, a new form of autosomal recessive early-onset parkinsonism (PARK20), due to mutations in the gene encoding the phosphoinositide phosphatase, Synaptojanin 1 (Synj1), has been reported. Several genes responsible for hereditary forms of Parkinson’s disease are implicated in distinct steps of the endolysosomal pathway. However, the nature and the degree of endocytic membrane trafficking impairment in early-onset parkinsonism remains elusive. Here, we show that depletion of *Synj1* causes drastic alterations of early endosomes, which become enlarged and more numerous, while it does not affect the morphology of late endosomes both in non-neuronal and neuronal cells. Moreover, Synj1 loss impairs the recycling of transferrin, while it does not alter the trafficking of the epidermal growth factor receptor. The ectopic expression of *Synj1* restores the functions of early endosomes, and rescues these trafficking defects in depleted cells. Importantly, the same alterations of early endosomal compartments and trafficking defects occur in fibroblasts of PARK20 patients. Our data indicate that Synj1 plays a crucial role in regulating the homeostasis and functions of early endosomal compartments in different cell types, and highlight defective cellular pathways in PARK20. In addition, they strengthen the link between endosomal trafficking and Parkinson’s disease.

## Introduction

Synaptojanin 1 (Synj1) is an inositol-phosphatase belonging to the family of Sac domain-containing proteins^1,2^. Remarkably, with respect to the other lipid phosphatases, Synj1 contains two distinct phosphatase domains: the Sac1 domain and the 5′-phosphatase domain^2,3^. The Sac1 domain of Synj1 predominantly dephosphorylates phosphatidylinositol (PI) monophosphates localised at Golgi and endosome membranes^3,4^, whereas the 5′-phosphatase domain dephosphorylates PI bi- or trisphosphates localised at the plasma membranes^2,5^. Hence, thanks to this double enzymatic activity, Synj1 is involved in different pathways depending on the cellular context^6^. So far, it has been shown that Synj1, together with its interacting partners dynamin and endophilin, is required for synaptic vesicle endocytosis^2,5,7,8^. Moreover, Synj1 appears to participate in the actin cytoskeleton polymerisation/depolymerisation events^9,10^. Recently, it has also been implicated to play a critical role in proper membrane trafficking in zebrafish cone photoreceptors^11,12^.

Parkinson’s disease (PD) is the second most common age-related progressive neurodegenerative disorder^13,14^. Although 90% of PD cases are idiopathic, at least 10% are inherited, and several causative genes have been identified^13–15^. Although these PD genes encode a functionally diverse set of proteins, many of them are implicated in several steps of the endolysosomal pathway^14,16^. However, the nature and degree of endocytic membrane trafficking impairment in early-onset parkinsonism remains to be elucidated^16^. Recently, mutations in the *Synj1* gene have been reported to be associated with PARK20^17–19^. The same homozygous mutation, R258Q, was identified independently in three families: one of Iranian and two of Italian origin^17–19^. Afterwards, a novel homozygous mutation (c.1376C>G, p.R459P) in *Synj1* was identified in an Indian family^20^. Both mutations are in the Sac1 domain. R258Q has been reported to abolish both 3- and 4-phosphatase activities, and do not affect the activity on PI(4,5)P_2_^18^.

To give further insights into the role of Synj1 in the control of endocytic pathways, we analysed the morphology and dynamics of endosomal trafficking in neuronal and non-neuronal cells in which the expression of *Synj1* was suppressed. We show that loss of Synj1 impairs vesicular trafficking at the plasma membrane/early endosome (EE) boundary. Remarkably, a similar loss of endosomal function was also revealed in primary cultures of fibroblasts derived from patients bearing the *Synj1* homozygous R258Q mutation, suggesting that defective endocytic trafficking might be implicated in PARK20 pathogenesis.

## Results

### The loss of Synj1 drastically impairs the homeostasis of EEs

To analyse the role of loss of Synj1 function on endosomal trafficking, we produced two human cell lines, HeLa and neuroblastoma-derived SH-SY5Y cells, in which the expression of *Synj1* was interfered by plasmid vectors encoding specific short hairpin RNAs (shRNAs; see Materials and methods section).

**Fig. 1:**
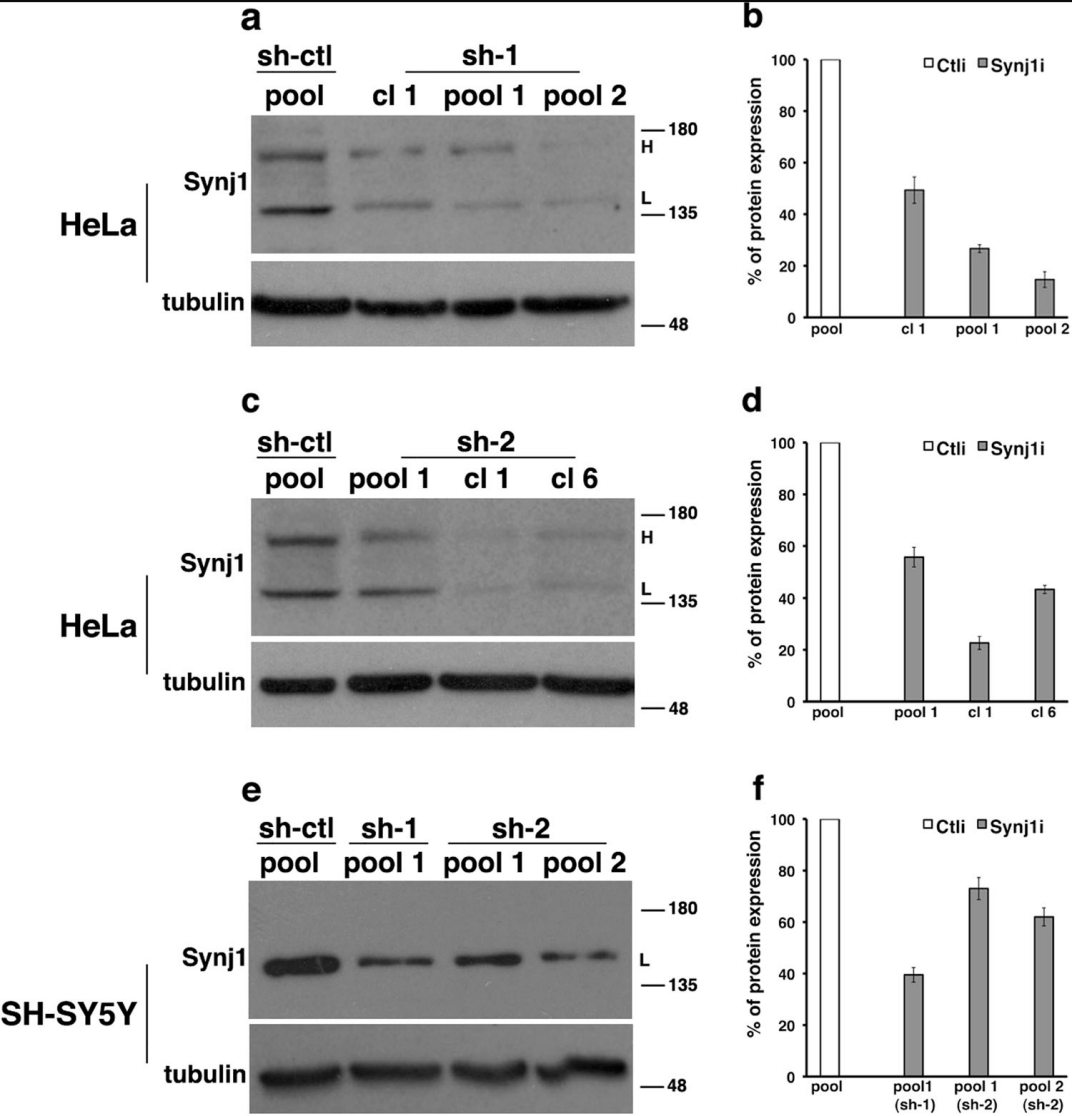
The expression of Synj1 was stably interfered in HeLa and SH-SY5Y cells by using short hairpin RNAs. HeLa **a**, **c** and SH-SY5Y **e** cells stably transfected with scrambled (sh-ctl) or specific anti-Synj1 (sh-1 and sh-2) shRNA were tested for the expression of Synj1 by western blotting. Tubulin was used as loading control. Representative immunoblotting is shown. The molecular weight of protein markers is indicated. H and L correspond to 170 and 145 kDa isoforms, respectively. As previously described^60^, only the 145 kDa isoform has been found in neuronal cells. **b**, **d**, **f** Densitometric analyses of three different experiments are shown. Results are expressed as mean values ± SD of the Synj1-interfered pool and clones compared with scrambled interfered cells (set equal to 100%)

For each cell line, several pool of clones and single clones (in the case of HeLa cells), ranging from 30 to 80% of silencing, were selected (Fig. 1), and those with a reduction of about 40–45% were used for further experiments. Remarkably, in all selected HeLa clones, the expression of *Synj1* shRNAs reduced the expression of both isoforms of the protein, 170 and 145 kDa (Figs. 1a-d).

**Fig. 2:**
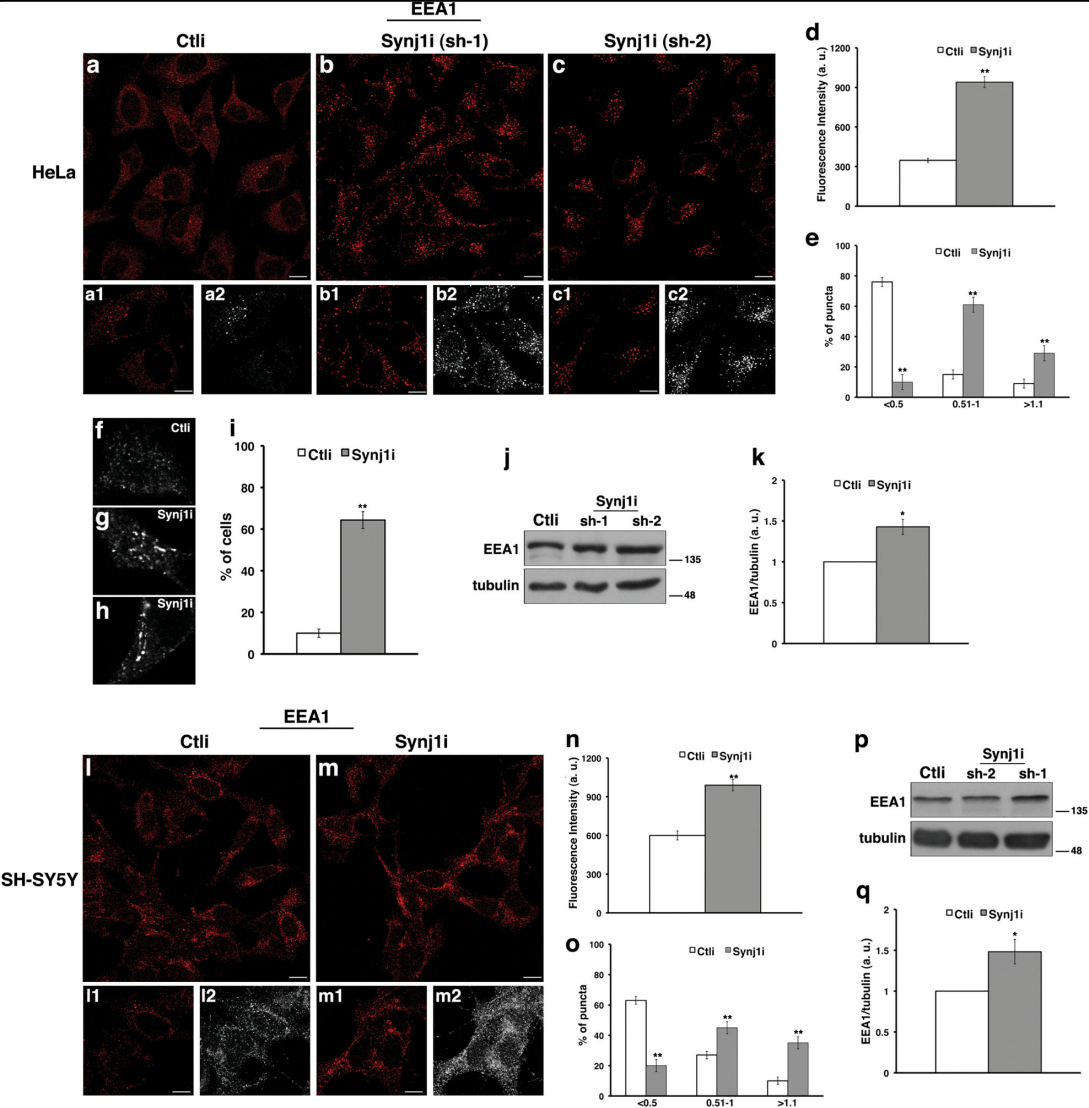
The loss of Synj1 drastically affects the homeostasis of early endosomes in HeLa and SH-SY5Y cells. Ctli and Synj1i HeLa **a-c**, **f-h** and SH-SY5Y **l**,** m** cells were stained with EEA1 (early endosome antigen 1) antibody detected with Alexa-546-conjugated secondary antibodies. Serial confocal sections were collected from the top to the bottom of the cells. **a-c** Representative images showing that the early endosomes are more abundant and enlarged in Synj1i than in Ctli HeLa cells. Scale bars, 10 μm. For each condition, pictures at higher magnification (**a1**-**c1**) and 3D reconstruction (**a2**-**c2**) are shown. Scale bars, 5 μm. The mean fluorescence intensity (**d**, arbitrary unit, a.u.) and the percentage **e** of larger EEA1-positive structures (>1.1 μm) are strongly increased in Synj1i than in Ctli cells. The bars show relative mean values ± SD of three independent experiments in four stably interfered HeLa cells (pool2 and cl1 for sh-1; pool1 and cl1 for sh-2), *n* ≥ 50 cells. **f-h** Representative 3D reconstruction images show a greater number of EEA1-positive tubular structures in Synj1i cells than controls. **i** The bars show mean values ± SD of three independent experiments in interfered HeLa cells (the aforementioned pools for both shRNAs); *n* ≥ 50 cells. **j** Representative immunoblotting of EEA1 in Ctli and Synj1i HeLa cells. Tubulin was used as loading control. The molecular weight of protein markers is indicated. **k** Densitometric analysis of three different experiments performed in stably interfered HeLa cells (same pools and clones as in e) is shown. **l**, **m** Representative images showing early endosomes more enlarged in Synj1i than in Ctli SH-SY5Y cells. Scale bars, 10 μm. For each condition, pictures at higher magnification (**l1**, **m1**) and 3D reconstruction (**l2**, **m2**) are shown. Scale bars, 5 μm. **n**, **o** Mean fluorescence intensity (arbitrary unit, a.u.) in Ctli and Synj1i SH-SY5Y cells is shown. Experiments were performed three independent times (using pool1 for sh-1, pool2 for sh-2), *n* ≥ 50 cells. **p**, **q** Immunoblot detection of EEA1 and densitometric analysis in Ctli and Synj1i SH-SY5Y pools as above described. Error bars, means ± SD. *p < 0.05 **p < 0.01, Student’s* t*-test

**Fig. 3:**
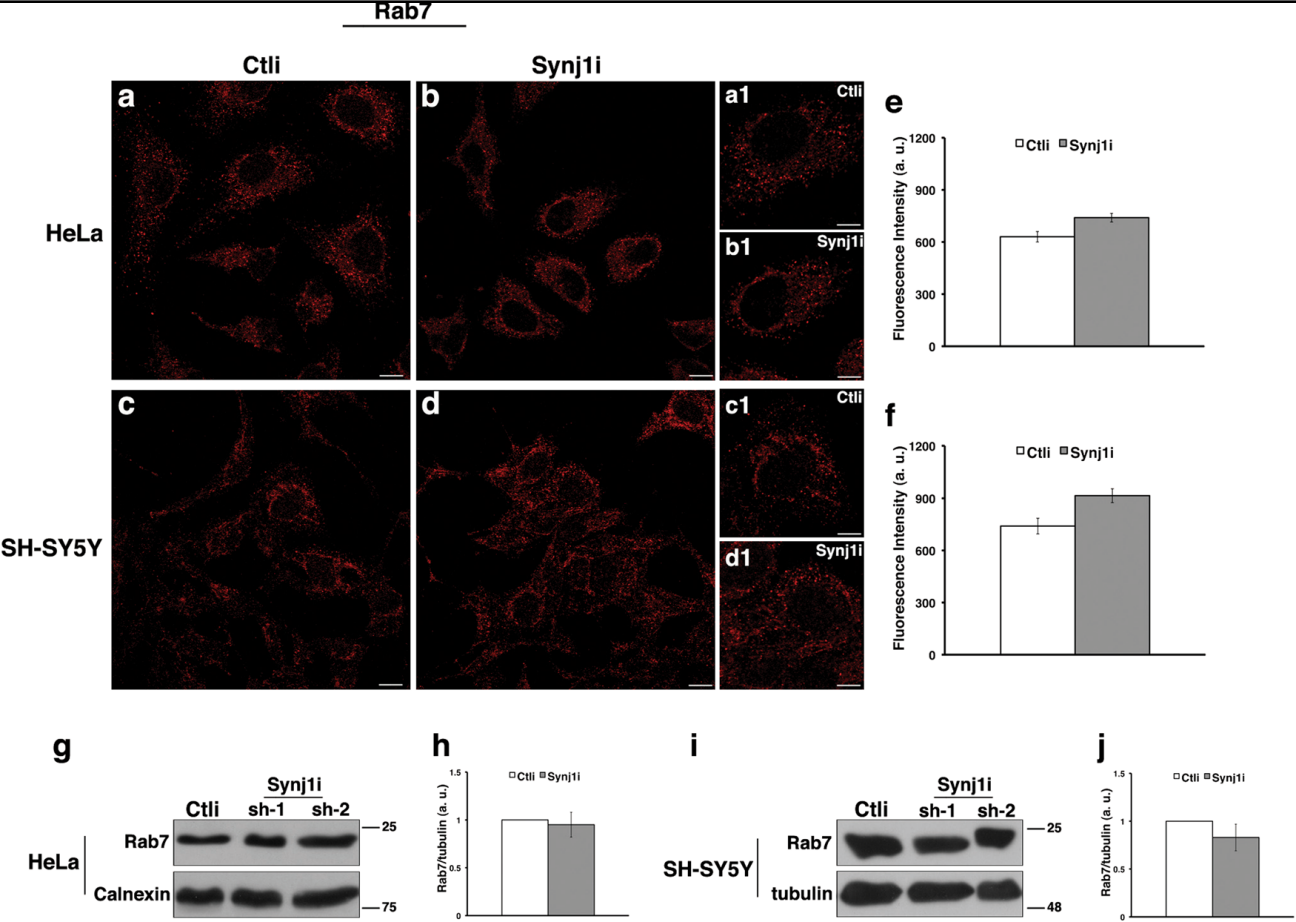
The loss of Synj1 does not alter the homeostasis of late endosomes in HeLa and SH-SY5Y cells. Ctli and Synj1i HeLa **a**, **b** and SH-SY5Y **c**,** d** cells were stained with Rab7 antibody detected with Alexa-546-conjugated secondary antibodies. Serial confocal sections were collected from the top to the bottom of the cells. Representative images showing comparable late endosomes in Ctli and Synj1i cells **a-d**. Scale bars, 10 μm. Pictures at higher magnification (**a1-d1**) are shown; scale bars, 5 μm. **e**, **f** Mean fluorescence intensity (arbitrary unit, a.u.) in Ctli and Synj1i cells is shown. The bars show relative mean values ± SD of three independent experiments performed in pools and clones as described in Figs. 2e and n; *n* ≥ 50 cells. **g-j** Immunoblot detection of Rab7 **g**, **i** and densitometric analyses **h**, **j** of three different experiments performed in aforementioned stably interfered HeLa and SH-SY5Y cells. Calnexin and tubulin were used as loading control. The molecular weight of protein markers is indicated. Error bars, means ± SD

To assess the potential impact of Synj1 deficiency on the endocytic pathway, we analysed the morphology and dynamics of the endolysosomal compartment in either Synj1-silenced cells (Synj1i) or scrambled RNA transfected control cells (Ctli) by immunofluorescence assays using different markers of the endocytic route (Figs. 2, 3).

We observed that the depletion of endogenous Synj1 causes striking changes of EE structures (Fig. 2). As revealed by the EEA1 antibody, the EEs increased in both number and size in HeLa (Figs. 2a-e) and SH-SY5Y (Figs. 2l-o) Synj1i cells when compared with EEs in Ctli cells. Strikingly, silenced HeLa cells displayed numerous EEA1-positive tubular structures (Figs. 2f-h), and the percentage of cells containing these structures was drastically higher in Synj1i than in Ctli cells (Fig. 2i). Furthermore, in both HeLa and SH-SY5Y Synj1i cells, the endosomal structures displayed higher fluorescence intensity with respect to control cells (Figs. 2d, e, n, o), indicating an enlargement of these compartments. Consistently, western blot analysis showed higher levels of EEA1 both in HeLa (Figs. 2j, k) and in SH-SY5Y (Figs. 2p, q) Synj1i with respect to Ctli cells, possibly due to its altered dynamics. In addition, we obtained comparable results for Rab5, another early endosomal marker, by immunofluorescence (Supplementary Figures S1a-f) and western blot (Supplementary Figures S1g-j) assays in both cell lines, thus providing further evidence for EE alterations.

Furthermore, to rule out potential “off-target” effect generated by shRNAs we analysed the morphology of EEs by immunofluorescence assays upon transient transfection of three different specific small interfering RNAs (siRNAs) against Synj1 in HeLa cells (Supplementary Figures S2a-j), confirming the aforementioned results.

In contrast, in both cells lines we found no major alterations of the late endosomes labelled with anti-Rab7 antibody both by immunofluorescence (Figs. 3a-f) and immunoblot (Figs. 3g-j) experiments. Similar results were obtained by staining early and late endocytic compartments of Ctli and Synj1i HeLa cells with GFP-Rab5 and GFP-Rab7, respectively (Supplementary Figure S3).

In general, our results indicate that the loss of Synj1 may exclusively alter the homeostasis of EEs in both HeLa and SH-SY5Y silenced cells, suggesting that Synj1 plays a similar role in the control of EE homeostasis in different cell types.

In addition, Synj1 was localised in proximity of plasma membrane as shown by double immunofluorescence assays with a secretory membrane protein, PrPc (Supplementary Figures S4a-c). Moreover, Synj1 was in close contact with EEA1-positive dots (Supplementary Figures S4d-g). These findings further support the hypothesis of its involvement in the control of endocytic functions.

### The loss of Synj1 affects recycling trafficking, but not trafficking to lysosomes

EEs represent an important sorting station for recycling back to plasma membrane internalised molecules or eventually for their targeting to the lysosome for degradation or transportation to early secretory compartments^21,22^.

Therefore, to determine whether the loss of Synj1 not only alters the morphology of EEs, but might also inhibit the functions of these organelles, we performed endocytosis assays in both the Synj1-silenced cells and in the scrambled shRNA-transfected cells. To this aim, we examined the endocytic pathway of the transferrin (Tf) receptor (TfR) and the epidermal growth factor (EGF) receptor (EGFR), which upon internalisation follow two different distinct itineraries: recycling and degradation, respectively^21,22^.

**Fig. 4:**
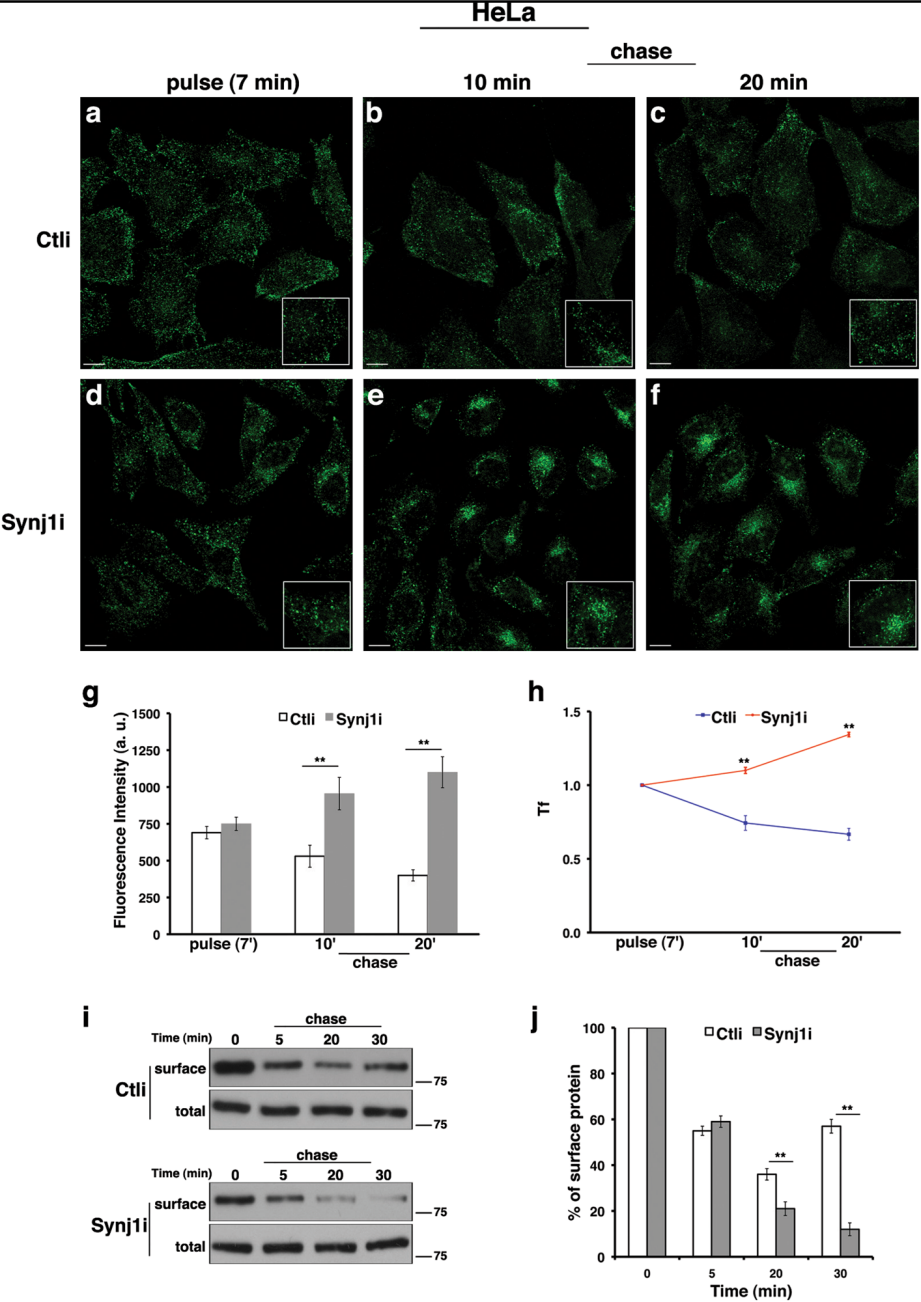
The loss of Synj1 leads to intracellular accumulation of transferrin in HeLa cells. (**a-h**) Internalisation assay of Alexa-488-conjugated transferrin (Tf) in Ctli and Synj1i HeLa cells (see also Materials and methods section). Briefly, Tf was added to the cells at 37 °C for 7 min (pulse; **a**, **d**). After washing out, cells were incubated in culture medium for different indicated times (chase; **b**, **c**, **e**, **f**). Representative single confocal sections **a-f** show that Tf is uptaken similarly in control and silenced cells, whereas it is more intracellularly stalled in *Synj1*-interfered cells. Bars, 10 μm. Higher magnification pictures are shown in the insets. 3D reconstructions are shown in Supplementary Figure S6a-f. **g** Mean fluorescence intensity (arbitrary unit, a.u.) of Tf is shown. Experiments were performed three independent times in different silenced cells (pool2 and cl1 for sh-1; pool1 for sh-2). Error bars, means ± SD; *n* ≥ 50 cells, **p < 0.01, Student’s *t*-test. (**h**) Curves of Tf internalisation expressed as mean values of fluorescence measured at chase times compared with the fluorescent signal after pulse (set to 1) are shown (*p* < 0.001, Bonferroni test after significant ANOVA). **i**, **j** Immunodetection of Tf receptor at the surface upon Tf induction by biotinylation assay (see Materials and methods section) in Ctli and Synj1i cells. Briefly, cells were incubated with Tf for 30 min at 4 °C to prevent its internalisation (time 0), washed and then warmed at 37 °C in culture medium. At the end of each chase time, cells were labelled with LC-biotin. One-tenth of lysates (total) was kept before streptavidin precipitation. Densitometric analysis is shown **j**, and the results were expressed as percentage of the amount of protein at the time 0. Error bars, means ± SD; **p < 0.01, Student’s* t*-test

**Fig. 5:**
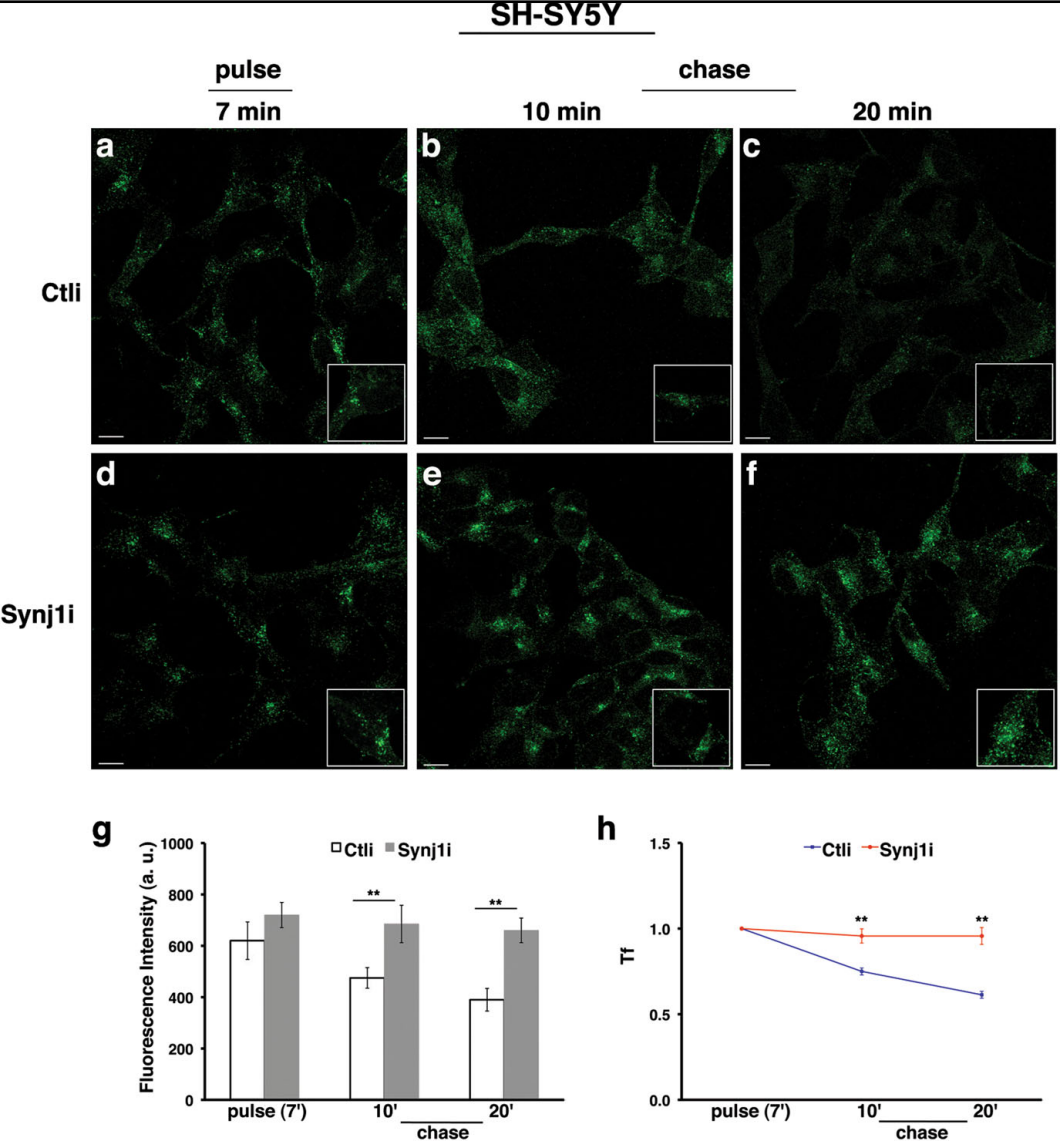
The loss of Synj1 leads to intracellular accumulation of transferrin in SH-SY5Y cells. **a-h** Internalisation assay of Alexa-488-conjugated transferrin (Tf) in Ctli and Synj1i SH-SY5Y cells. Cells were pulsed with Tf for 7 min and then chased for different indicated times as described in Fig. 4. Representative single confocal sections show that Tf is uptaken similarly in control and silenced cells, whereas it is more intracellularly stalled in *Synj1*-interfered cells. Bars, 10 μm. Higher magnification pictures are shown in the insets. 3D reconstructions are shown in Supplementary Figure S6g-l. **g** Mean fluorescence intensity (arbitrary unit, a.u.) of Tf is shown. Experiments were performed three independent times in the two aforementioned pools. Error bars, means ± SD; *n* ≥ 50 cells, **p < 0.01, Student’s *t-*test. **h** Curves of Tf internalisation expressed as mean values of fluorescence measured at chase times compared with the fluorescent signal after pulse (set to 1) are shown (*p* < 0.001, Bonferroni test after significant ANOVA)

To monitor the trafficking of TfR, cells were incubated with Alexa Fluor-488 or -546-conjugated Tf at 37 °C for different time periods (5, 10, and 30 min). At 5 min of internalisation, the signal of Tf was comparable between control and silenced cells (Supplementary Figure S5a and c,e), indicating that the loss of Synj1 does not affect its uptake and endocytosis. Conversely, at later time points, a strong increase (of about 2.5-fold) of Tf signal was observed in Synj1i cells (Supplementary Figure S5b and d-e), indicating that Tf accumulates intracellularly and, on the other hand, that its recycling to the surface is impaired. Strikingly, in Synj1-depleted cells 30 min after internalisation, Tf-positive compartments were mislocalised in the paranuclear region (Supplementary Figure S5b and d). A similar result was obtained when HeLa and SH-SY5Y cells were incubated with Tf at 37 °C for 7 min (pulse), washed to remove the unbound Tf, and then incubated at 37 °C for different time periods in the culture medium (chase; Figs. 4a-h and 5a-h). In these experimental conditions, we found no difference in the fluorescent signal and pattern in Ctli and Synj1i cells upon pulse (Figs. 4a, d and 5a, d), confirming that the absence of Synj1 does not interfere with Tf internalisation. As expected, we observed a progressive decrease in Tf signal at the chase times, both in HeLa (Figs. 4b, c, g, h) and SH-SY5Y (Figs. 5b, c, g, h) control cells. On the contrary, in Synj1i-depleted cells the fluorescent signal remained higher at the same chase time points (Figs. 4e-h and 5e-h), indicating that Tf is intracellularly stalled. The specificity of Tf trafficking alterations is supported by the finding that the same effect was observed upon siRNAs transient transfection (Supplementary Figures S2k-t). To confirm these results, we monitored the amount of TfR at the surface after Tf exposure (30 min at 4 °C, time 0) for different chase times by biotinylation assays (Figs. 4i, j). Whereas at later time points, the extent of biotinylated TfR increased in Ctli cells, it remained low in Synj1i cells (Figs. 4i, j), indicating that recycling of the receptor is impaired upon Synj1 silencing.

**Fig. 6:**
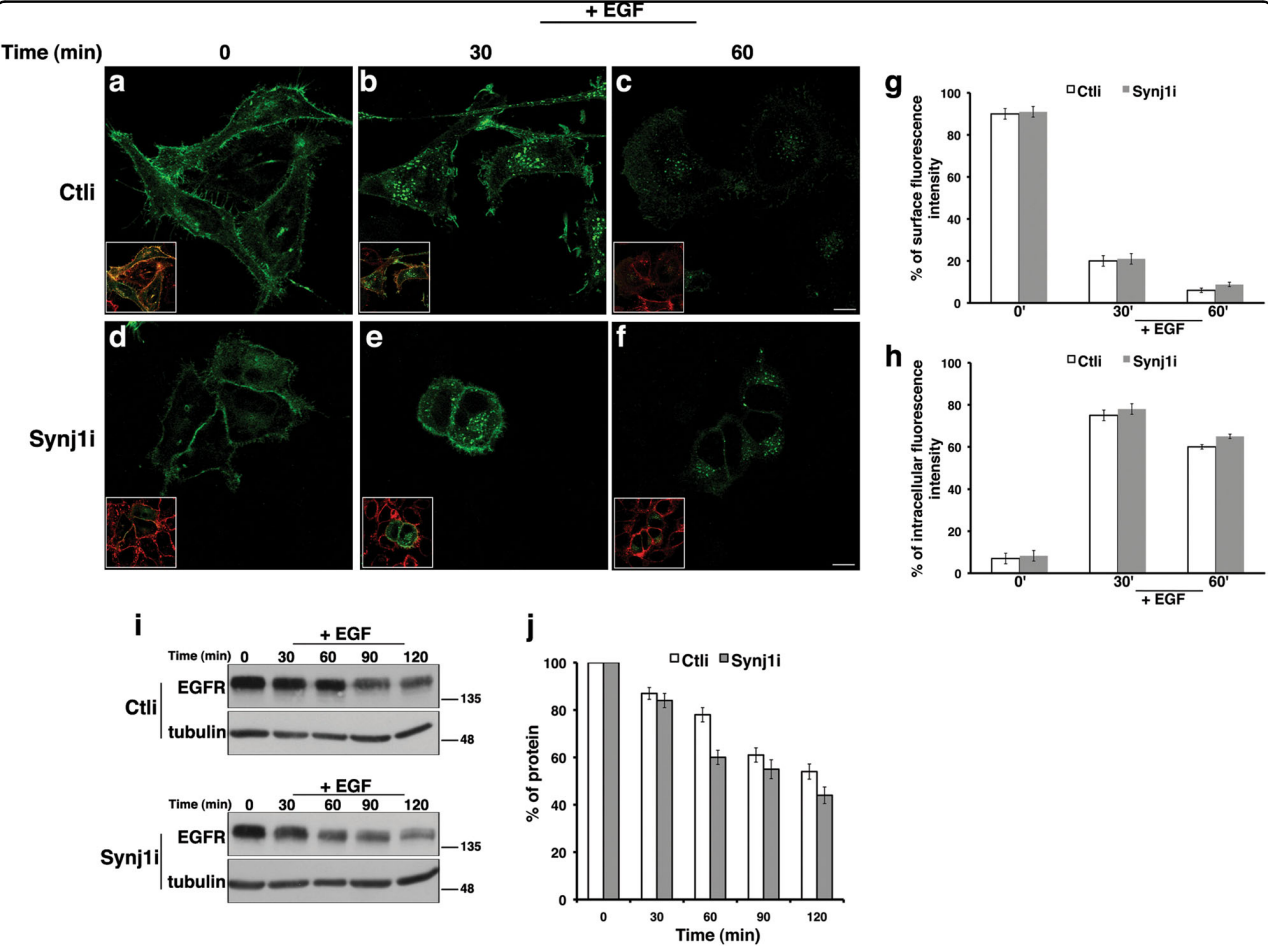
The loss of Synj1 does not alter the trafficking of EGF receptor upon EGF binding in HeLa cells. **a-h** Ctli and Synj1i HeLa cells were transiently transfected with cDNA coding for GFP–EGFR. 48 h after transfection, cells were serum starved for 3 h (time 0; **a**, **d**), incubated with EGF (100 ng/ml) for different indicated times **b**, **c**, **e**,** f**, fixed and stained with a plasma membrane marker, CD55 (red). Serial confocal sections were acquired from the top to the bottom of cells. The overlay of green and red fluorescence is shown in insets. Representative images show that EGFR follows a comparable kinetic of internalisation upon EGF binding in Ctli and Synj1i cells. Bars, 10 μm. **g**,** h** Mean fluorescence intensities at the surface **g** and in the intracellular compartments **h** were measured at the different chase times and expressed as percentages of total fluorescence. **i**, **j** Representative immunoblotting of EGFR upon EGF stimulation. Cells were treated as aforementioned, and after each chase time they were lysed and subjected to western blot analysis **i**. Tubulin was used as loading control. The molecular weight of protein markers is indicated. Densitometric analysis is shown **j**, and the results were expressed as percentage of the amount of protein at the time 0. Error bars, means ± SD

To further examine the effect of Synj1 inactivation on endosomal trafficking, we analysed the dynamics of the EGFR in the Ctli and Synj1i cells. For this purpose, Ctli or Synj1i HeLa cells were transiently transfected with a plasmid vector bearing the complementary DNA (cDNA) coding for the green fluorescent protein (GFP)–EGFR fusion protein. As previously described^23^, 48 h after transfection, cells were serum starved for 3 h in order to prevent ligand-dependent internalisation. As expected, following starvation (time 0), a higher amount of the GFP–EGFR protein was found at the cell surface (Figs. 6a, d). The GFP–EGFR protein entered the cells after EGF stimulation (100 ng/mL) with a kinetic comparable between control and silenced cells (Figs. 6a-h), strongly indicating that trafficking towards the lysosomes was not affected in the Synj1-deficient cells. Moreover, we evaluated EGFR levels upon EGF induction at different times in presence of cycloheximide (Figs. 6i, j). We observed no differences between Ctli and Synj1i cells, further suggesting that EGFR degradation is unaffected by Synj1 silencing.

Conclusively, these results clearly indicate that Synj1 is crucial for the recycling pathway in the early endosomal compartment.

### The loss of Synj1 slightly alters morphology of lysosomes

**Fig. 7:**
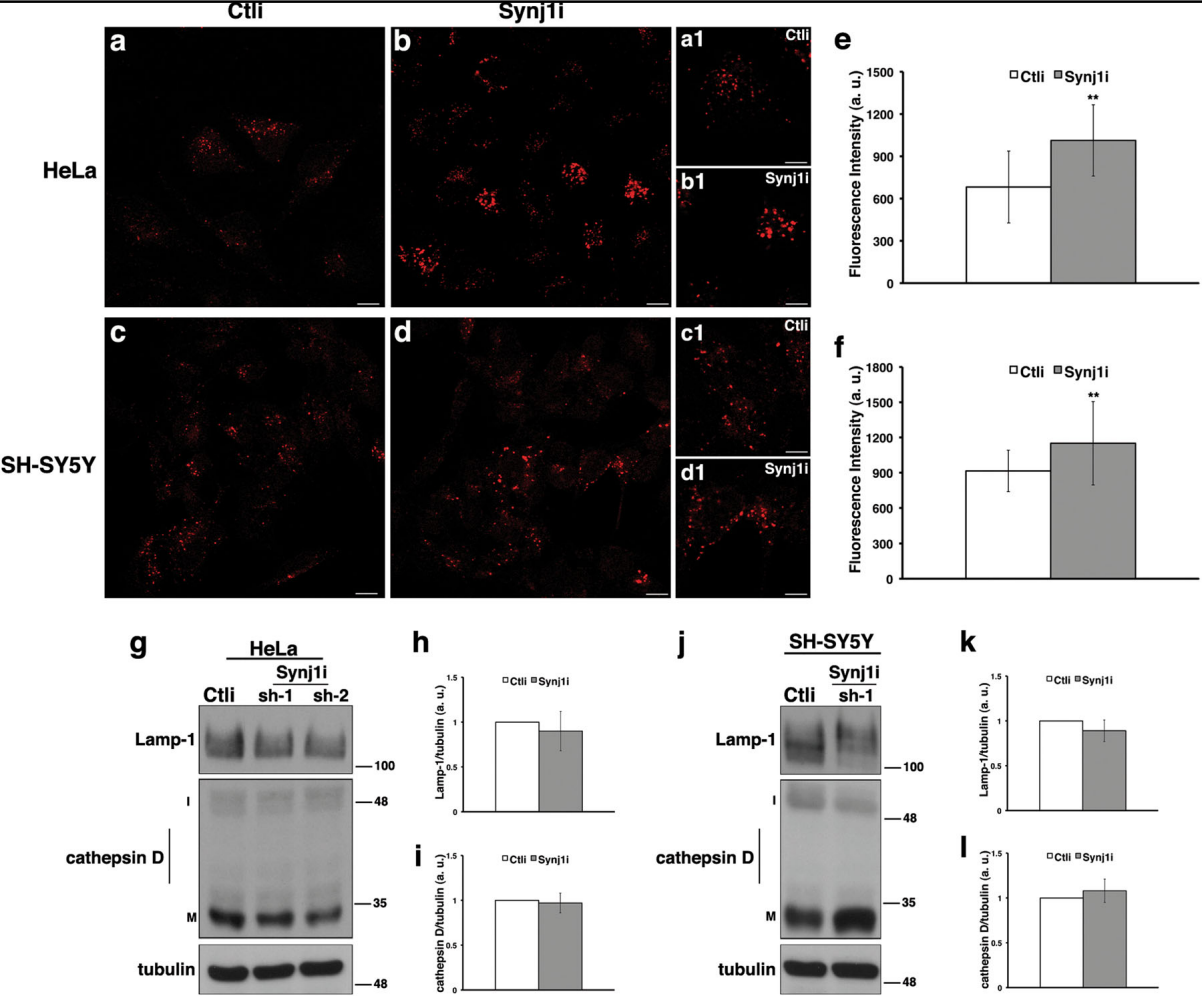
The loss of Synj1 slightly affects the lysosomal compartments in HeLa and SH-SY5Y cells. **a-d** Lysosomes of Ctli and Synj1i HeLa **a**,** b** and SH-SY5Y **c**, **d** cells were labelled by using Lysotracker dye, which was added to living cells for 1 h at 37 °C (see Materials and methods section). Serial confocal sections were collected from the top to the bottom of the cells. Representative images showing a slight alteration of these organelles in Synj1i compared with Ctli cells **a-d**. Scale bars, 10 μm. Higher magnification pictures are shown in the insets (**a1-d1**). Bars, 5 μm. **e**, **f** Mean fluorescence intensity (arbitrary unit, a.u.) in Ctli and Synj1i cells is shown. Experiments were performed three independent times in different silenced clones as aforementioned. Error bars, means ± SD; *n* ≥ 50 cells. **g-l** Representative immunoblotting of Lamp-1 and cathepsin D in Ctli and Synj1i HeLa **g** and SH-SY5Y **j** cells and densitometric analysis (**h**,** i** and **k**,** l** for HeLa and SH-SY5Y, respectively). Tubulin was used as loading control. The molecular weight of protein markers is indicated. Mature (M; 33 kDa) and immature (I; 52 kDa) forms of cathepsin D are shown. Error bars, means ± SD. **p < 0.01 Student’s *t*-test

Furthermore, to investigate whether the loss of Synj1 could affect the late steps of endocytic pathway, we analysed the morphology of the lysosomal compartments. As shown by lysotracker dye labelling (Figs. 7a-f), in the major part of HeLa and SH-SY5Y-silenced cells, lysosomes resulted enlarged and appeared as fluorescent dots with more intense signal, indicating an alteration of these organelles. Comparable results were observed for the lysosomal associated membrane protein 1 (Lamp-1) in both cell lines (Supplementary Figures S7a-f). No significant differences of two lysosomal markers, Lamp-1 and cathepsin D, were detected by western blot analyses (Figs. 7g-l). Overall, all these findings indicate the Synj1 silencing slight affects lysosomal compartments.

### The re-expression of Synj1 restores proper functions of early endosomal compartments

**Fig. 8:**
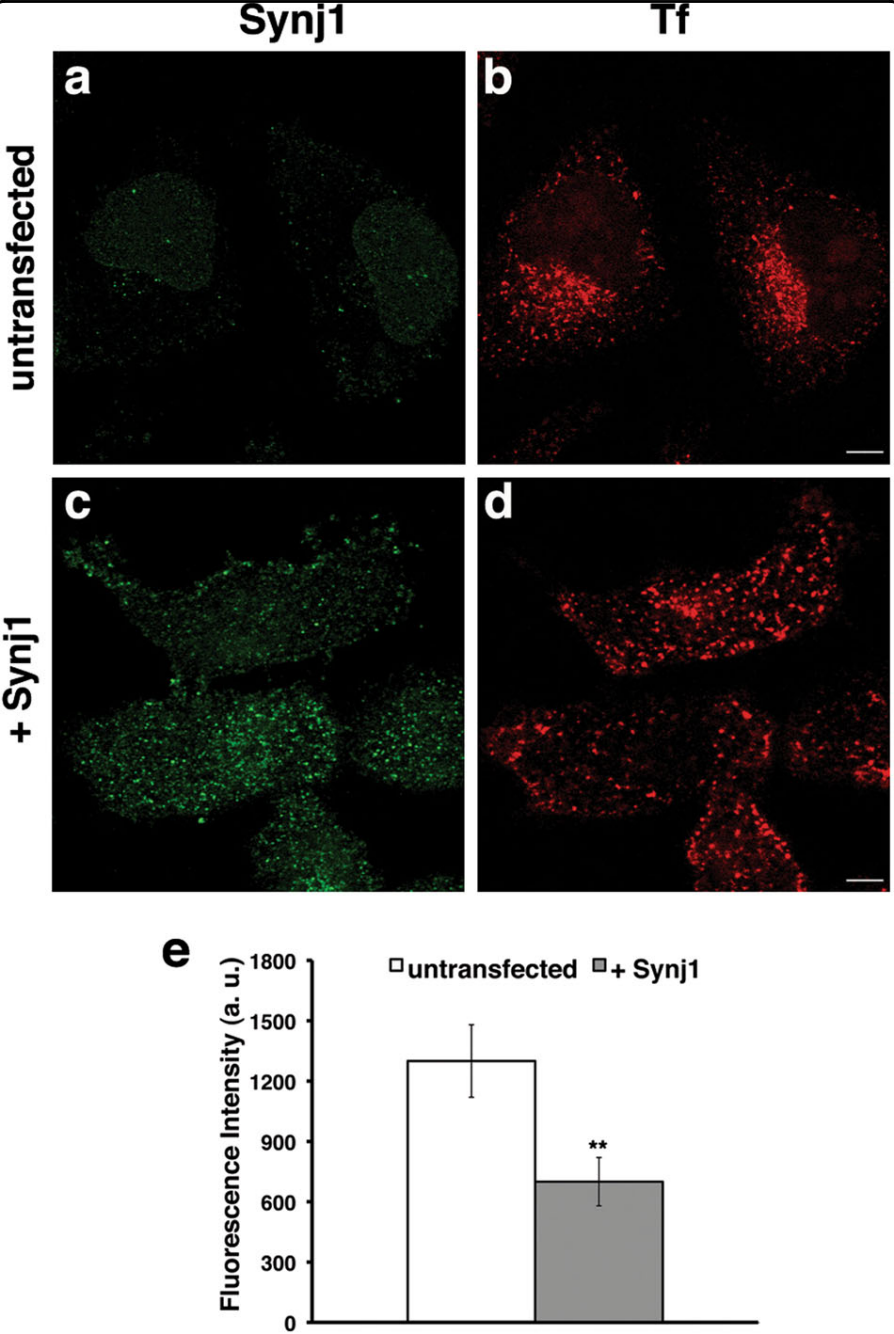
Ectopic expression of wild-type Synj1 restores the proper trafficking of transferrin. **a-d** Synj1i HeLa were transiently transfected with cDNA coding for wild-type Synj1 ( + Synj1, **c, d**) or untransfected (just electroporated, **a, b**). Forty-eight hours after transfection, cells were subjected to a Tf internalisation assay as described in Fig. 4. After fixation, cells were stained with a specific antibody anti-Synj1 (green). Representative images corresponding to 20 min chase of Tf **a-d** and mean fluorescence intensity (arbitrary unit, a.u.; **e**) in untransfected and transfected cells are shown. Error bars, means ± SD, *n* ≥ 30 cells; **p < 0.01, Student’s *t*-test. Scale bars, 5 μm

To rule out the possibility that the effects observed on the endosomal pathway is not directly linked to Synj1, we transiently transfected Synj1-depleted HeLa cells with a plasmid vector encoding the wild-type Synj1 protein. In these experimental conditions, we assessed the internalisation of Tf by performing pulse-chase experiments as described in Fig. 4. Differently to silenced cells, no intracellular accumulation of Tf was found in Synj1-transfected cells after 20 min of chase (Fig. 8), indicating that the re-expression of Synj1 restores proper trafficking of Tf.

These data confirm the critical role of Synj1 in regulating the functions of early endosomal compartments.

### Patient fibroblasts show enlarged EEs and an impairment of recycling trafficking

All the data obtained in the knocked-down cells indicate that Synj1 is crucial for the homeostasis and functions of EEs in different human cell types. Hence, to further corroborate our hypothesis, we assessed the morphology of EEs and the recycling trafficking in fibroblasts of PARK20 patients^17,19^.

**Fig. 9:**
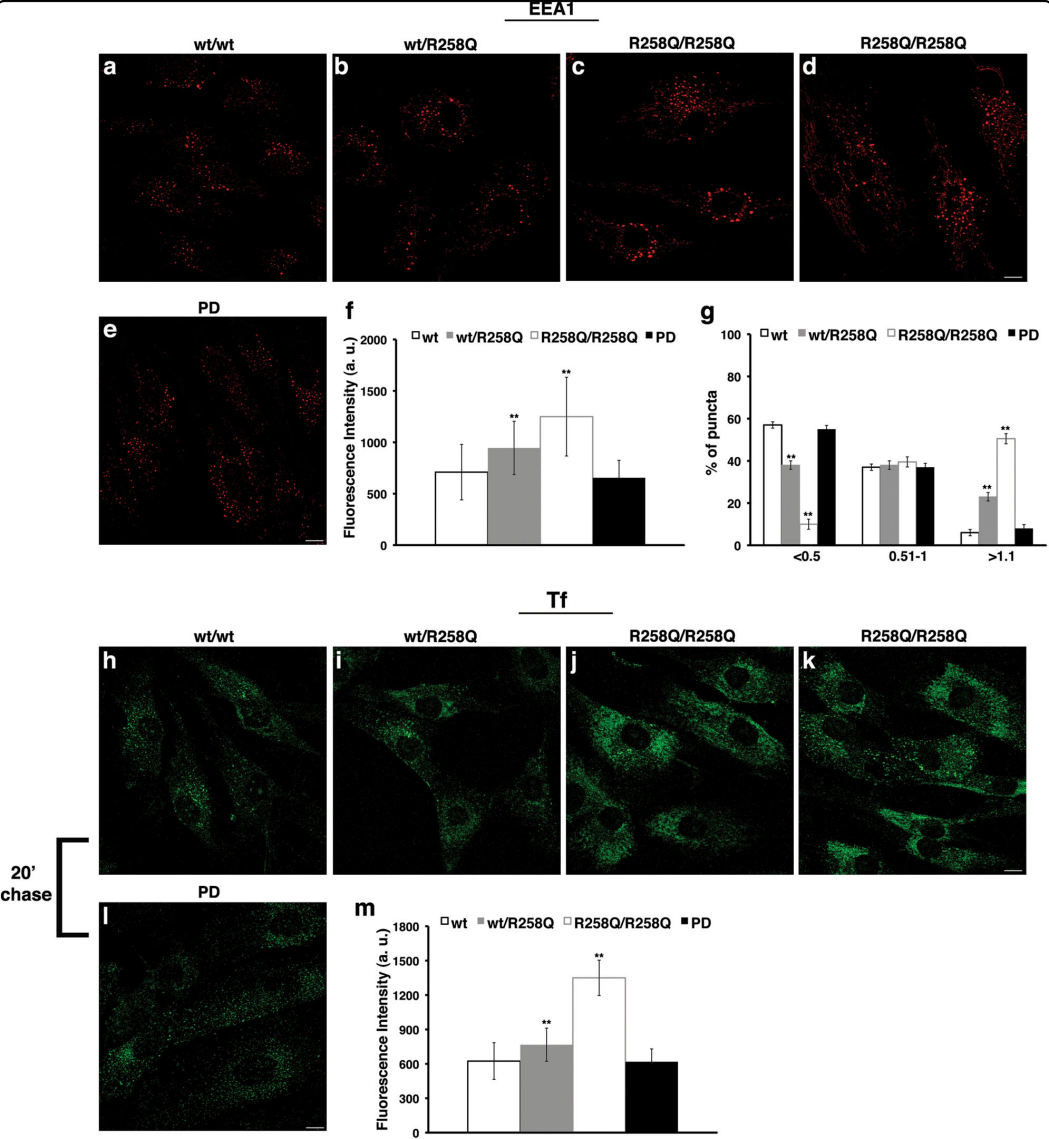
The homeostasis of early endosomes and transferrin trafficking is altered in patient fibroblasts. **a-e** Early endosomes of fibroblasts from health control (wt/wt), heterozygous R258Q carrier (wt/R258Q), homozygous PARK20 (R258Q/R258Q) and idiopathic PD patients were labelled with EEA1, detected with Alexa-546-conjugated secondary antibodies. Serial confocal sections were collected from the top to the bottom of the cells. Scale bars, 5 μm. **f**,** g** The mean fluorescence intensity (arbitrary unit, a.u.; **f**) and the percentage of larger EEA1-positive structures (>1.1 μm; **g**) were significantly higher in affected individuals than in control or PD fibroblasts. The bars show mean values ± SD of three independent experiments performed in two individuals per each condition; *n* ≥ 50 cells. **p < 0.001, Bonferroni test after significant ANOVA. **h-m** Fibroblasts were subjected to a Tf internalisation assay as described in Fig. 4. Representative images corresponding to 20-min chase of Tf **h-l** and mean fluorescence intensity (arbitrary unit, a.u.; **m**) are shown. Scale bars, 10 μm. The bars show mean values ± SD of three independent experiments performed in two individuals per each condition; *n* ≥ 50 cells. **p < 0.001, Bonferroni test after significant ANOVA

In agreement with the results obtained in silenced cells, we found that in the fibroblasts of patients, EEs were expanded, as shown by the presence of larger and more intense fluorescent spots (Figs. 9a-g). Moreover, Tf was greatly accumulated inside the cells of patients versus fibroblasts from healthy controls and idiopathic PD patients, confirming that its recycling was impaired (Figs. 9h-m). Interestingly, the fibroblasts from healthy heterozygous p.R258Q carriers displayed an intermediate phenotype (Figs. 9b, i) because they showed, for both analysed parameters (EEA1 staining and Tf internalisation), a statistically significant difference with respect to both controls and PARK20 fibroblasts (Figs. 9f-g, m).

Altogether these results indicate that alteration of homeostasis and function of early endosomal compartments is Synj1 dependent and might be involved in PARK20 neurodegeneration and pathogenesis.

## Discussion

The mechanistic link between Synj1 and early-onset parkinsonism is still unknown. This study demonstrates that Synj1 plays a crucial role in the homeostasis and function of EEs in different cell types, including neuronal cells.

### Defects of Synj1 loss

We showed that upon Synj1 depletion EEs, but not late endosomes, are enlarged and, sometimes, mislocalised in the paranuclear region. Moreover, although the internalisation of different ligands, such as Tf or EGF, is unaffected in Synj1-depleted cells, the loss of Synj1 impairs the recycling of the TfR to the plasma membrane. In contrast, it does not alter the trafficking of EGFR to the lysosomes, indicating that the recycling pathway is specifically affected by the lack of Synj1. Altogether, these data unravel a novel role for Synj1 in regulating membrane trafficking in human cells. Consistently, a fraction of Synj1 is recruited to EEs, as shown by its partial colocalisation with EEA1.

Although in presynaptic terminals, the role of Synj1 in synaptic vesicle endocytosis and recycling is well established^2,5,7,24–26^, only few studies have reported an involvement of Synj1 in membrane trafficking. Loss of Synj1 has been shown to lead to defects in the endolysosomal pathway in photoreceptor neurons of zebrafish^12,27^. These defects include enlarged acidic vesicles, abnormal late endosomes, an increase in autophagosomes, as well as an abnormal accumulation of synaptic proteins within photoreceptor cell bodies^12,27^. Contrary to this, our observations clearly highlight that, in human cells, Synj1 is required for controlling the homeostasis of EEs and their sorting functions. Consistently, the expression of the DNAJC13 mutant, associated with autosomal dominant PD, leads to accumulation of Tf in endosomal compartments, also affecting their morphology^28^. Moreover, mutations in other proteins associated with PD have been shown to alter functions of these compartments^29–31^. Furthermore, the accumulation of abnormal early endosomal structures have also been reported in the cell bodies of neurons from mice overexpressing *Synj1*^32^, indicating that the level of expression of this phosphatase has to be very finely tuned.

Interestingly, rod photoreceptors from zebrafish mutants lacking *Synj1*, with respect to cones develop normally and do not display morphological defects^11^, thus postulating a differential role of Synj1 in these cell types. However, Synj1 is ubiquitous and in humans it has a broad tissue distribution, where it is expressed at comparable levels. Differently to what occurs in other species, we found similar defects in endosomal trafficking in human neuronal and non-neuronal cells, clearly indicating its ubiquitous role in human cells.

Together with previous findings, our data provide clear evidence that Synj1 plays a pivotal role in regulating membrane trafficking, not only at the synaptic terminals, where it is required for synaptic vesicle reavailability^5,7,26,33^, but also in the cell body of neurons, as in other cell types where it is crucial for endosomal recycling.

Furthermore, we also observed an enlargement of lysosomes in Synj1-silenced cells. This could be a consequence of the altered membrane trafficking from EEs. However, this hypothesis is not supported by the fact that late endosomes are not altered upon Synj1 loss, as the trafficking of EGFR to lysosomes and its lysosomal degradation appear to be unaffected. On the other hand, the alteration of lysosomes could be due to increased levels of autophagy. According to this hypothesis, a role for Synj1 in regulating autophagy has been proposed in zebrafish cone photoreceptors^27^ as in flies^34^. Moreover, in an Alzheimer’s mouse model, altering Synj1 expression causes changes in the delivery of amyloid beta to lysosomes^35^. Whether Synj1 might play a similar role in mammalian cells and whether this could be concurrent with the pathogenesis of PD will have to be explored in future studies.

How the loss of Synj1 might alter EE homeostasis and function? It has long been known that phosphoinositides (phosphorylated derivatives of PI) are essential components of cellular membranes and, thanks to the versatility of the inositol group, have been implicated in many fundamental biological processes^36–39^. In the last decade, they have emerged as important regulators of membrane trafficking^36,40–42^. Because of their differential subcellular distribution, phosphoinositides might allow the selective recruitment of proteins containing PI recognition modules to specific membrane compartments. Thus, the levels of phosphoinostides must be finely regulated in time and space, and this is achieved by strict control of the subcellular distribution, membrane association and activity state of kinases and phosphatases.

Thanks to two consecutive phosphatase domains, Sac1 and 5′-phosphatase, Synj1 mainly dephosphorylates PI bi- or trisphosphates localised in plasma membranes, and PI monophosphates, PI(4)P and PI(3)P, enriched in the membranes of Golgi apparatus and endosomes, respectively^36^. For this peculiarity, Synj1 might be responsible of different functions. The conversion of PI(4,5)P_2_ to PI(4)P has been shown to be required for the clathrin uncoating and vesicle endocytosis^5,7^. Although the deficiency of PI(4)P might affect the structure and functions of the Golgi complex^43^, its role in the plasma membrane is still unclear, except for an indirect action as a crucial substrate to generate PI(4,5)P_2_. It has been shown that PI(3)P contributes to controlling the function of EEs as sorting stations in the biosynthetic and the endocytic pathways^36,42^. Moreover, a variety of endosomal proteins contain PI(3)P-binding modules, such as EEA1 and Hrs (hepatocyte growth factor-regulated tyrosine kinase substrate)^44–46^, and therefore the proper recruitment, both in spatial and temporal terms, to these compartments through PI binding could be crucial to exert their functions. Thus, it is likely that the imbalance of PI(3)P levels might lead to the alteration of EEs. Among endosomal proteins, it is conceivable that the dysfunction of EEA1, which is a Rab5 effector and is required for endosomal tethering, could be responsible for the observed defects. Further studies will be needed to elucidate this aspect.

### Defects of the Synj1 mutation

Our immunofluorescence studies and endocytosis assays revealed that EEs of PARK20 fibroblasts with the homozygous missense R258Q are enlarged and the recycling of Tf is impaired in these cells. Fibroblasts of healthy R258Q heterozygous carriers show minor alterations in endosome trafficking and recycling. These findings suggest that alterations of homeostasis and functions of EEs may account for the occurrence of the disease and that endosomal trafficking is related principally to the Sac1 domain. In agreement with this, it has been shown that the R258Q mutation abolishes both 3- and 4-phosphatase activities raised by the Sac1 domain, whereas it does not affect the activity on PI(4,5)P_2_^18^. Previous studies showed that both enzymatic domains contribute to synaptic vesicle recycling in mice and in nematode worms^7,25,47^, although the loss of 5′ phosphatase activity had more severe consequences than the loss of Sac1 domain activity^7^. Moreover, the 5′ phosphatase, but not the Sac1 domain of Synj1, has been described as being involved in regulating endolysosomal and autophagic trafficking in zebrafish cones^12,27^.

In contrast, our data from fibroblasts of patients and healthy R258Q carriers suggest that the Sac1 domain is necessary for proper endosomal trafficking, and its activity should be at least 50% to guarantee correct functionality.

### Role of endosomal pathways in PD

Much evidence has highlighted that vesicle trafficking pathways are implicated in PD mechanisms. In particular, current emerging data highlight the pivotal role of the autophagy–lysosome pathway in PD pathogenesis^14,16,48–50^. Mutations in ATP6AP2, responsible of a X-linked parkinsonism with spasticity, reduce the activity of the vacuolar (H^+^)-ATPase proton pump compromising lysosomal acidification^51^. The expression of mutated leucine-rich repeat kinase 2 (LRRK2), associated with PARK8, delays the degradative trafficking of EGFR and causes enlarged lysosomes^52,53^. These defects may be ascribed to the decreased levels of active Rab5 (due to enhanced kinase activity) with reduced early to late endosome maturation^54^. The zinc pump ATP13A2, mutated in PARK9, localises to multivesicular bodies and has been found to promote the extracellular release of alpha-synuclein via exosomes^55–57^. Hence, through different mechanisms, these PD proteins might lead to the dysfunction of these pathways, with consequent impairment of lysosomal degradation, therefore resulting in protein accumulation (among them alpha-synuclein) and neurotoxicity. On the other hand, the endosomal system, at crossroads of distinct intracellular pathways, is a fundamental sorting station and is essential for the maintenance of cellular homeostasis. In the last few years, mutations in VPS35 (PARK17) and VPS26A (both components of retromer complex) and in the endosomal DNAJC13 (PARK21) have been associated with familial and sporadic forms of PD^28–31^, suggesting that the dysfunction of this critical cellular hub is a pathological mechanism of disease. Our data provide new evidence for the implication of EEs in PD, and together with past observations, emphasise the role of endosomal trafficking in the pathogenesis of this disease.

Regarding other cell types, neurons are more dependent on these pathways, to ensure the fine balance between recycling and degradation of synaptic proteins and/or of specific cargoes, such as neurotransmitters or growth factor receptors, as well as to supply bulk membrane flow required for the continuous turnover of neuritis^48^. Our observations that in the SH-SY5Y cells the altered endosomal trafficking was exhibited by a lower degree of *Synj1* silencing and the number of knock down surviving cells was lower compared with non-neuronal cells support the hypothesis that neurons are more susceptible to the dysfunction of these pathways with respect to other cell types. In addition, this phenomenon may help to explain the central nervous system tissue-specific susceptibility of PARK20.

We can hypothesise that the dysfunction of early endocytic compartments correlates with the alteration of neuronal plasticity or with the loss of neuronal viability. A diffuse, nonspecific brain atrophy was observed only in the Sicilian family by MRI, whereas a marked decrease of dopamine transporter density in the striatum has been found in all the patients subjected to single-photon emission computed tomography with intravenous injection of 185 MBq of [^123^I]FP-CIT (DaT-SCAN)^17,19^. These data suggest that the defective EEs might lead to dysfunction of neuronal functions. A recent study showing that neurons of mice carrying the R258Q mutation also displayed endocytic defects and a delay in synaptic vesicle endocytosis upon electrical stimulation^26^ further supports this hypothesis. However, we cannot exclude that the dysfunction of these compartments could also affect cell survival over time and this can correlate with the progression of the disease. Further studies will be important to elucidate these aspects.

## Materials and methods

### Reagents and antibodies

Primary antibodies include the following: mouse monoclonal anti-EEA1 (Abcam) and rabbit polyclonal anti-EEA1 (ThermoFisher Scientific) for western blotting, monoclonal rabbit anti-EGF receptor (Cell Signalling), goat polyclonal antibody anti-cathepsin D (Santa Cruz), mouse monoclonal anti-Lamp-1 (BD Phamigen); mouse monoclonal anti-Rab7 (Santa Cruz); mouse monoclonal anti-Rab5 (BD Transduction Laboratories); rabbit polyclonal anti-Synj1 (Abcam); mouse monoclonal anti-Tf receptor (ThermoFisher Scientific). LysoTracker® Red DND-99 and fluorescent Tf conjugates were from Molecular Probes (Invitrogen). Alexa Fluor secondary antibodies were from Life Technologies, and horseradish peroxidase (HRP)-conjugated secondary antibodies used for western blot analysis were from GE Healthcare. Basic chemicals were from Sigma-Aldrich or AppliChem GmBH.

### Cell cultures

#### Cell lines

HeLa and SH-SY5Y cells were maintained in RPMI-1640 with 10% fetal bovine serum (FBS), and 2 mM l-glutamine. All cells lines were maintained at 37 °C in a saturated humidity atmosphere containing 95% air and 5% CO_2_.

#### Human fibroblasts

Fibroblasts from two patients (p.R258Q/p.R258Q) and two heterozygous carriers (p.R258Q/wt) from the two Italian PARK20 families were obtained after culturing skin punch biopsies^17,19^. Written informed consent was obtained from all the patients.

As controls, we used fibroblast cell lines derived from two healthy, age-/gender-matched individuals and two idiopathic PD patients. These cells were obtained from our institutional biobank. All cells were investigated at similar culture passages (P4–P6).

Cells were grown in Dulbecco’s modified Eagle’s medium (DMEM) supplemented with 2 mM glutamine, 10% FBS (PAA Laboratories GmbH, Pasching, Austria), and penicillin/streptomycin, at 37 °C and 5% CO_2_.

### Cell transfection and RNA interference

RNA interference was obtained by transfecting specific shRNAs (from Open Biosystems) inserted in pShag Magic version 2.0 (pSM2c) vector: shRNA-1 5′-TGAACATATGCTAAGTAAAT-3′; shRNA-2 5′-AAATACTCTGAATAGTGATT-3′. As negative control, we used an shRNA against GFP, 5′-GGCACAAGCTGGAGTACAACTA-3′.

Transfection was performed using Lipofectamine 2000 (Invitrogen) according to the manufacturer’s protocol. Stably transfected cells were obtained after selection with puromycin (0.6 μg/ml, Sigma). In particular, we collected both pool of clones or single clones derived from single-cell colonies. For most experiments, both pool of clones or single clones were used to better validate the effect of Synj1 silencing.

### Western blot analysis

Cells, grown on 100 mm Petri dishes, were lysed with JS lysis buffer (Hepes pH 7.5 50 mM, NaCl 150 mM, glycerol 1%, Triton X-100 1%, MgCl_2_ 1.5 mM, EGTA 5 mM) containing a cocktail of protease inhibitors (Sigma). Lysates were run on sodium dodecyl sulfate–polyacrylamide gel electrophoresis, transferred onto polyvinylidene difluoride or nitrocellulose membranes and revealed by western blotting using specific antibodies.

### Fluorescence microscopy

Cells, grown on coverslips, were washed with phosphate-buffered saline (PBS), fixed with 4% paraformaldehyde (PFA) and quenched with 50 mM NH_4_Cl. Then, cells were permeabilised with 0.2% Triton X-100 for 5 or 7 min (depending on the antibody) and blocked for 30 min in PBS containing 10% FBS and 1% bovine serum albumin (BSA). Primary antibodies were detected with Alexa Fluor-conjugated secondary antibodies.

For lysosome staining, cells were incubated for 1 h with Lysotracker (1:1000) in complete medium before fixing. Images were collected using a laser scanning confocal microscope (LSM 510; Carl Zeiss MicroImaging, Inc.) equipped with a planapo 63× oil-immersion (NA 1.4) objective lens by using the appropriate laser lines. Images were acquired with the confocal pinhole set to one Airy unit, taking Z-slices from the top to the bottom of the cell by using the same setting (laser power, detector gain), as well as the same threshold of fluorescence intensity in all experimental conditions (control and silenced cells). Quantification and colocalisation analyses were carried out using LSM 510 software as previously described^58,59^. The mean fluorescence intensities were measured by drawing regions of interest (ROIs) around the entire cell and corrected for background. The number and size of fluorescent puncta were carried out by using ImageJ software^59^. For GFP–EGFR internalisation experiments, the mean fluorescence intensity of surface and intracellular GFP signals was measured by drawing ROIs around plasma membrane labelled by a specific marker (CD55) and around areas, which excluded surface signals, respectively.

### Internalisation assays

#### Tf internalisation assay

To monitor Tf internalisation and recycling, we used two approaches. In the first approach, Alexa Fluor-488 or -546-conjugated Tf (10 μg/ml) was added to the cells in culture medium containing 1% BSA at 37 °C for different time periods (5, 10, 15, or 30 min). Then cells were fixed with 4% PFA.

In an alternative approach, cells were incubated with Tf for 7 min at 37 °C (pulse), washed to remove the excess of Tf and chased for different times (10, 20, and 30 min).

#### EGFR internalisation assay

The cells were transiently transfected with cDNA coding for the chimeric protein EGFR–GFP (kind gift of C. Puri) and after 48 h, were serum starved for 3 h as previously described^23^. Then, cells were stimulated with EGF (100 ng/ml) at 37 °C and fixed with 4% PFA for different times as indicated. In order to analyse only the trafficking of protein coming from surface avoiding newly protein synthesis, cells were incubated with cycloheximide (150 μg/ml) during the last hour of starvation and chase times. The same experimental procedure was followed for biochemical studies. In this case, after each time point cells were lysed and subjected to western blot analysis.

### Biotinylation assay

To detect the amount of Tf receptor at the surface upon Tf stimulation, biotinylation assay was carried out. First, cells were incubated with Tf for 30 min at 4 °C to prevent its internalisation (time 0). After washing to remove the excess of Tf, cells were incubated in culture medium for different chase times. Then, cells were biotinylated using LC-biotin (Pierce), lysed and precipitated with agarose–streptavidin beads (Pierce). Precipitated samples were revealed by western blotting using specific anti-TfR antibody.

### Statistical analysis

Two-tailed Student’s *t*-test or one-way analysis of variance (ANOVA) followed by Bonferroni multiple comparison test were used for statistical analysis when appropriate.

## Electronic supplementary material

Supplemental information

Figure S1

Figure S2

Figure S3

Figure S4

Figure S5

Figure S6

Figure S7
