# Supplementary material for: Alteration of endosomal trafficking is associated with early-onset parkinsonism caused by SYNJ1 mutations
Source: Cell Death Dis. 2018 Mar 7;9(3):385. doi: 10.1038/s41419-018-0410-7 (PMC5841278; doi:10.1038/s41419-018-0410-7)
Supplement: Supplementary file 1 — Supplemental information [file 41419_2018_410_MOESM1_ESM.pdf]

SUPPLEMENTARY INFORMATION

Supplementary figure legends

Figure S1: The loss of Synj1 affects the localization and levels of Rab5 in HeLa and SH-SY5Y cells. (**a-d**) Ctli and Synj1i HeLa (**a,b**) and SH-SY5Y (**c,d**) cells were labelled with Rab5 antibody detected with Alexa-546 conjugated secondary antibodies. Representative images show alteration of early endosomes in Synj1i compared to Ctli cells (**a-d**). Serial confocal sections were collected from the top to the bottom of the cells. Scale bars, 10 μm. Higher magnification pictures are shown in the insets (**a1-d1**). Bars, 5 μm. (**e,f**) Mean fluorescence intensity (arbitrary unit, a.u.) in Ctli and Synj1i cells is shown. Experiments were performed three independent times in different silenced clones (pool1, pool2 and cl1 for HeLa cells; pool1 and 2 for SH-SY5Y). Error bars, means ± SD; n≥50 cells. ** $p<0.01$, Student's t-test. (**g-j**) Representative immunoblotting of Rab5 (**g,i**) and densitometric analysis (**h,j**) performed in stably interfered HeLa and SH-SY5Y cells as above. Tubulin was used as loading control. The molecular weight of protein markers is indicated. Error bars, means ± SD. * $p<0.05$, Student's t-test.

Figure S2: Transient silencing of Synj1 affects the homeostasis of early endosomes and alters the transferrin recycling.

(**a**) qPCR analysis of Synj1 expression upon knockdown of Synj1. HeLa cells were transfected with three specific and different siRNAs for Synj1 (#1, #2, #3) and a control siRNA. After 72 hours from transfection the cells were collected and the level of Synj1 was measured by real time PCR. Data represent the mean of three independent experiments ± standard error mean (SEM) and are expressed as the fold changes relative to value measured in the cells transfected with control siRNA (siCtl). *$p<0.05$, Student's t-test. (**b-J**) HeLa cells were transiently transfected with control or three specific Synj1 siRNA and 72 hours later were stained with EEA1 antibody detected with Alexa-546

conjugated secondary antibodies. Representative images showing that the early endosomes resulted enlarged in Synj1i-deficient with respect to siCTR-interfered cells (**b-i**). Serial confocal sections were collected from the top to the bottom of the cells. Scale bars, 5 μm. 3D reconstructions are shown in f-i. (**j**) Mean fluorescence intensity (arbitrary unit, a.u.) is shown. Experiments were performed three independent times (n≥50 cells). Error bars, means ± SD. *p<0.01, Student's t-test. (**k-t**) Internalisation assay of Alexa-546 conjugated transferrin (Tf) as described in Figure 4. Representative single confocal sections show that Tf is uptaken similarly in control and silenced cells (7 min pulse, **k-n**), while it is more intracellularly accumulated after 20 min chase in Synj1-deficient cells than in control interfered cells (**o-r**). Bars, 10 μm. (**s,t**) The bars show mean fluorescence intensity (arbitrary unit, a.u.) of Tf after 7 min pulse (**s**) and 20 min chase (**t**) in the different Synj1-silenced cells relative to control interfered cells (set equal to 1). Experiments were performed three independent times. Error bars, means ± SD; n≥50 cells, ** p<0.01, Student's t-test.

Figure S3: The morphology of GFP-Rab5, but not GFP-Rab7 positive endosomes is affected upon Synj1 loss in HeLa cells. (**a-j**) Ctli and Synj1i HeLa cells were transiently transfected with cDNA coding for GFP-Rab5 (**a-d**) or GFP-Rab7 (**e-h**). After 48 hours from transfection, cells were fixed and images were acquired by confocal microscopy. Representative images corresponding to single confocal sections (**a, c, e, g**) and to 3D reconstructions (**b, d, f, h**) and the mean fluorescence intensity (**i,j**) are shown. Scale bars, 5 μm. Error bars show relative mean values ± SD of three independent experiments; n≥30 cells, ** p<0.01, Student's t-test; a.u., arbritary unit.

Figure S4: Synj1 partially co-localises with EEA1 in SH-SY5Y cells.

(**a-g**) SH-SY5Y cells were co-stained with specific antibodies anti-Synj1 and PrPc (plasma membrane marker; **a-c**) or EEA1 (early endosomal marker; **d-f**). Synj1 localised in cytoplasmic punctate structures, of which some were in proximity of plasma membrane (see arrows), other were

co-localised with EEA1-positive dots (see arrows). Scale bars, 5 μm. (**g**) The extent of overlap between Synj1 and EEA1 is represented by Pearson's coefficient. Error bar, mean ± SD of three independent experiments; n≥30 cells.

Figure S5: The loss of Synj1 leads to intracellular accumulation of transferrin in HeLa cells. **(a,e)** Internalisation assays of transferrin (Tf) in Ctli and Synj1i HeLa cells are shown. Cells were incubated with Alexa-488 conjugated Tf at 37°C for different times (5, 10, 30 min) and fixed. Representative images corresponding to 5 and 30 min of Tf addition (**a-d**) and mean fluorescence intensity (arbitrary unit, a.u.; **e**) in Ctli and Synj1i cells are shown. Scale bars, 10 μm. Higher magnification pictures are shown (**a1-d1**); scale bars, 5 μm. The bars show mean values ± SD of three independent experiments in two stably interfered HeLa cells; n≥50 cells, **p<0.01, Student's t-test. In contrast to control cells, in Synj1i cells Tf progressively accumulates upon internalisation, indicating that its recycling is affected by Synj1 loss.

Figure S6: The loss of Synj1 leads to intracellular accumulation of transferrin in HeLa cells. **(a-l)** 3D reconstructions of Ctli and Synj1i Hela (**a-f**) and SH-SY5Y (**g-l**) cells corresponding to single confocal sections shown in Figure 4 and 5, respectively. Insets show higher magnification pictures.

Figure S7: The loss of Synj1 slightly affects the lysosomal compartments in HeLa and SH-SY5Y cells. (**a-d**) Ctli and Synj1i HeLa (**a,b**) and SH-SY5Y (**c,d**) cells were labelled with Lamp-1 antibody detected with Alexa-546 conjugated secondary antibodies. Serial confocal sections were collected from the top to the bottom of the cells. Representative images showing a slight alteration of these organelles in Synj1i compared to Ctli cells. Scale bars, 10 μm. Higher magnification pictures are shown in the insets (**a1-d1**). Bars, 5 μm. (**e,f**) Mean fluorescence intensities (arbitrary

unit, a.u.) in Ctli and Synj1i cells are shown. Experiments were performed three independent times in different silenced clones (pool1, pool2 and cl1 for HeLa cells; pool1 and 2 for SH-SY5Y). Error bars, means ± SD; n≥50 cells. * $p<0.05$, ** $p<0.01$, Student's t-test.

Supplementary Methods

RNA extraction and quantitative PCR

For quantitative PCR (qPCR), total RNA was extracted by TriSure (Bioline). First-strand cDNA was synthesized using Mu-MLV RT (New England BioLabs) according to the manufacturer's instructions. qPCR was carried out with the QuantStudio 7 Flex (Thermo Fisher Scientific) using Fast SYBR Green PCR Master Mix (Thermo Fisher Scientific). The housekeeping GAPDH mRNA was used as an internal standard for normalization. qPCR data are presented as fold changes relative to the indicated reference sample using 2DeltaCt comparative analysis. Gene-specific primers used for amplification are listed below.

Synj1-f: 5'-CAACCCGATACCATCGGACA-3'

Synj1-r: 5'- TGTTTCCAGAGATCTCCCCG-3'

GAPDH-f: 5'- GTCGGAGTCAACGGATTTGG-3'

GAPDH-r: 5'- AAAAGCAGCCCTGGTGACC-3'
