# Supplementary figures and images for: Alteration of endosomal trafficking is associated with early-onset parkinsonism caused by SYNJ1 mutations
Source: Cell Death Dis. 2018 Mar 7;9(3):385. doi: 10.1038/s41419-018-0410-7 (PMC5841278; doi:10.1038/s41419-018-0410-7)

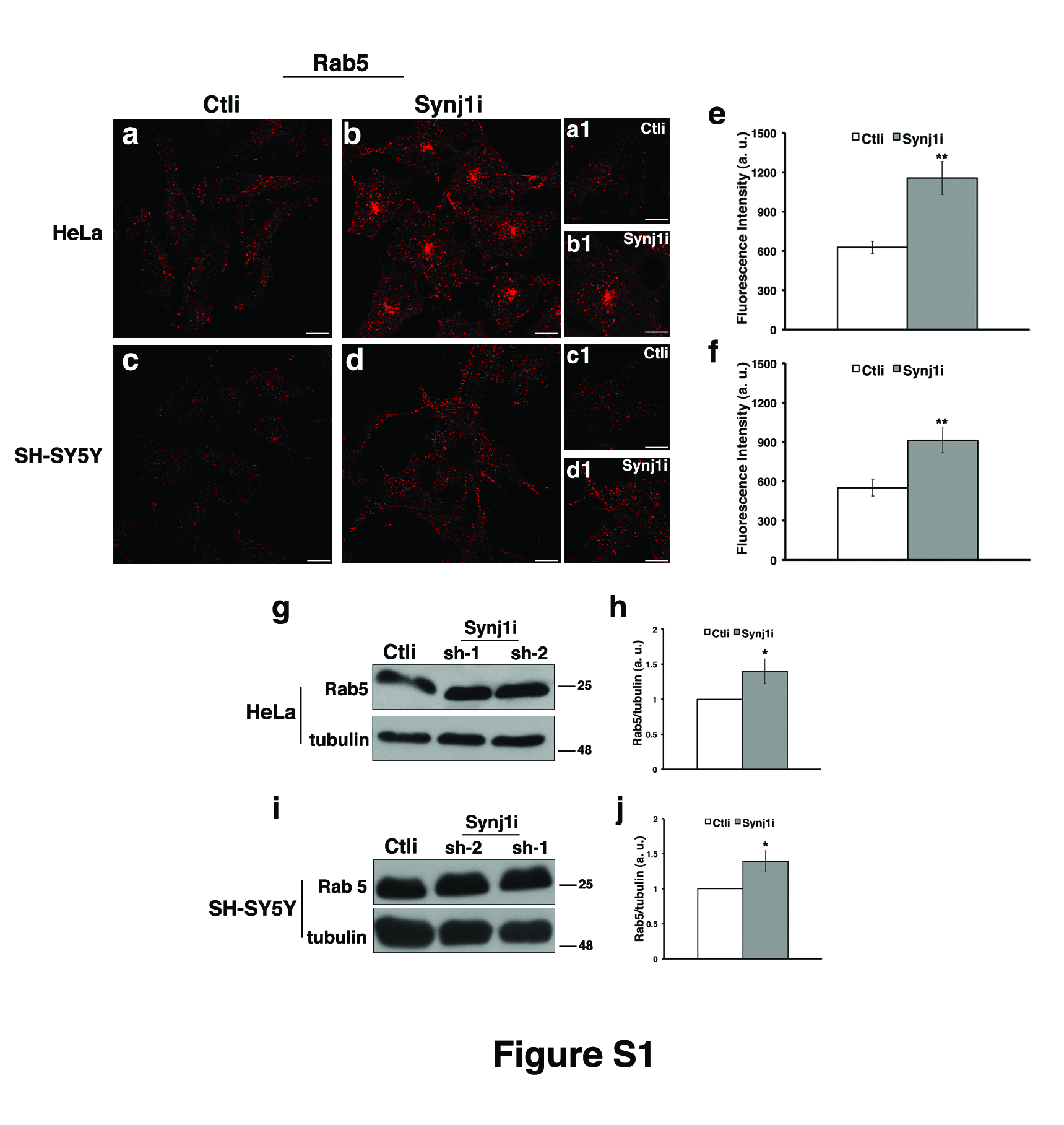

Supplement: Supplementary file 2 — Figure S1 [file 41419_2018_410_MOESM2_ESM.tif]

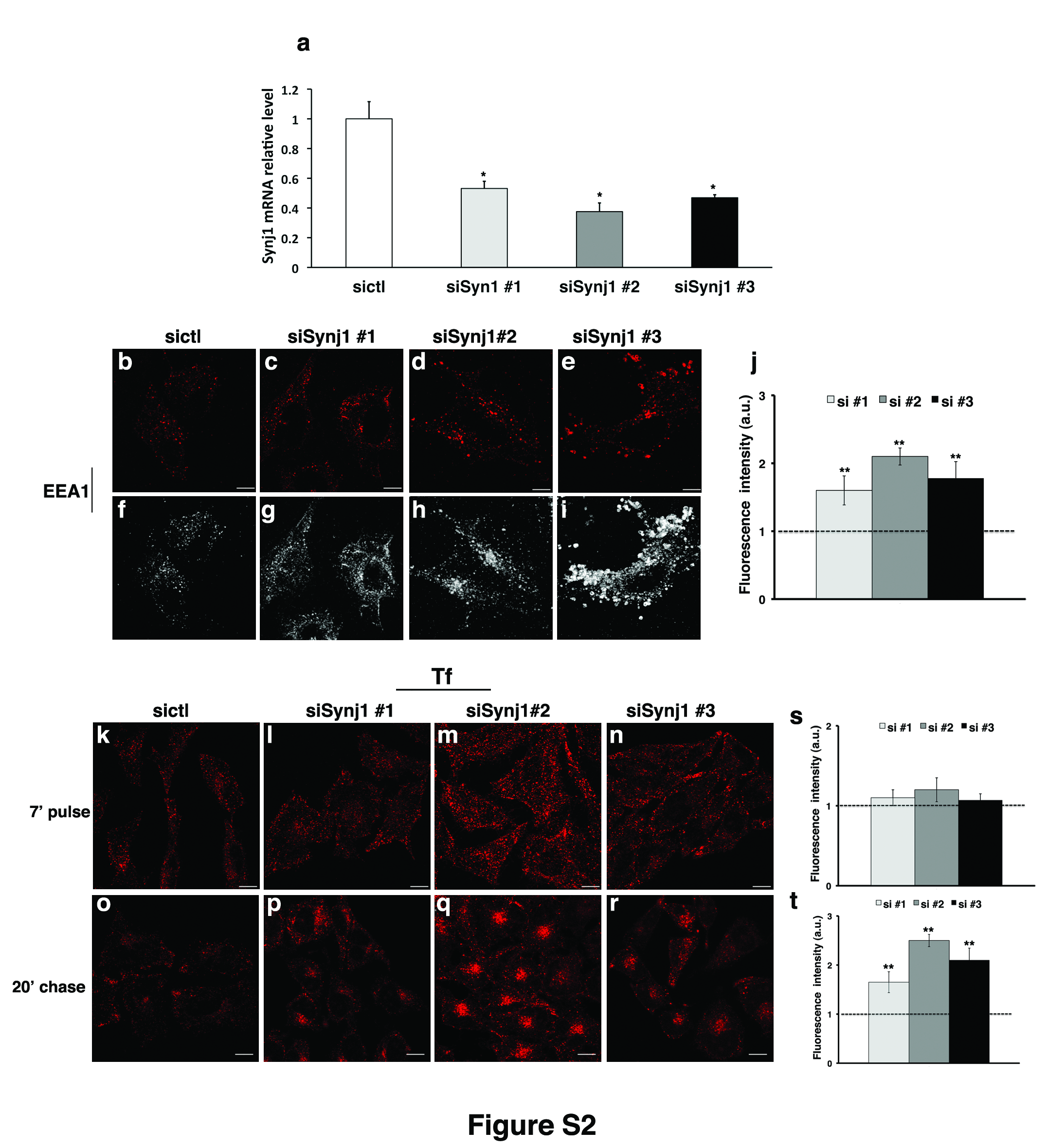

Supplement: Supplementary file 3 — Figure S2 [file 41419_2018_410_MOESM3_ESM.tif]

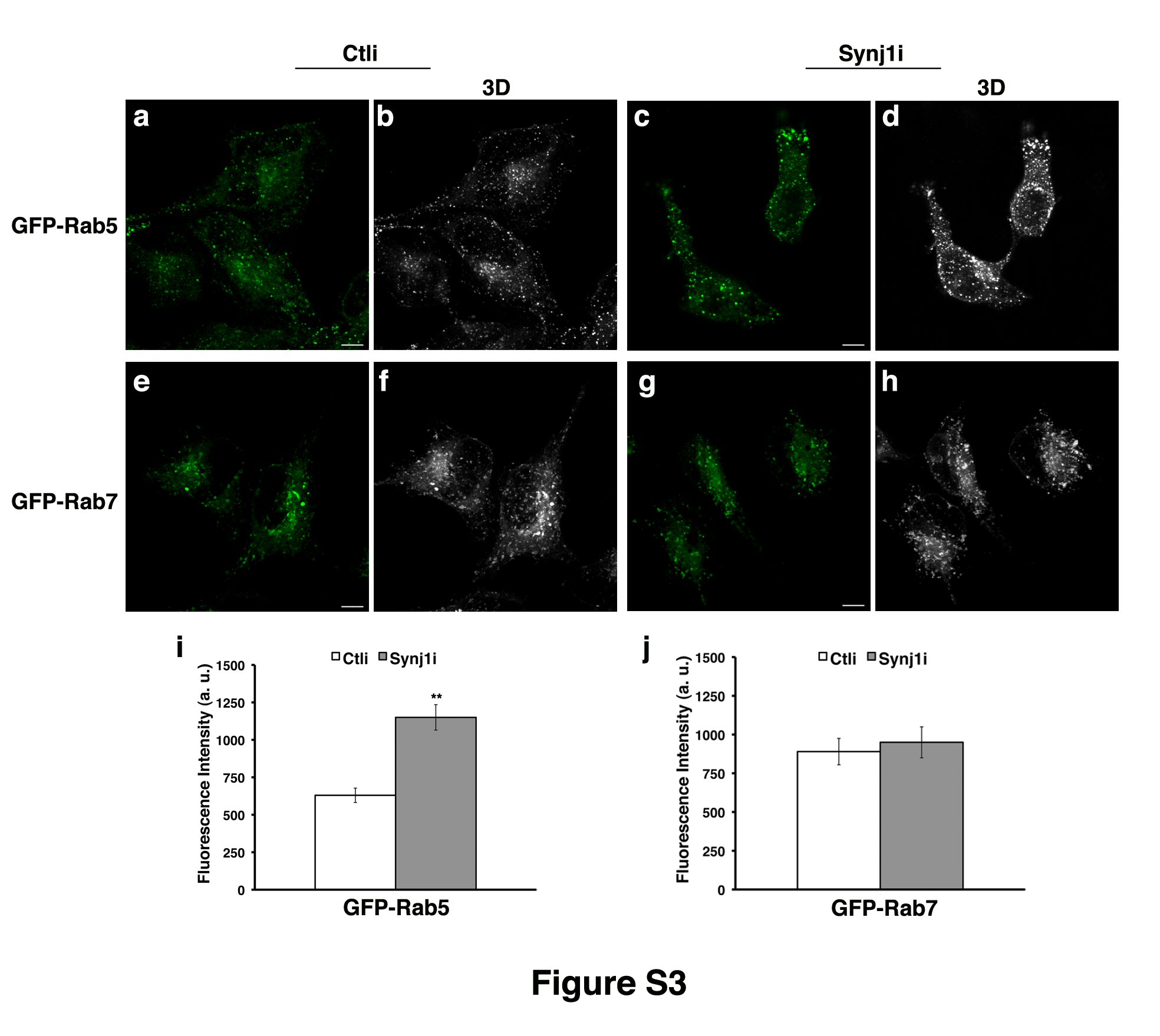

Supplement: Supplementary file 4 — Figure S3 [file 41419_2018_410_MOESM4_ESM.tif]

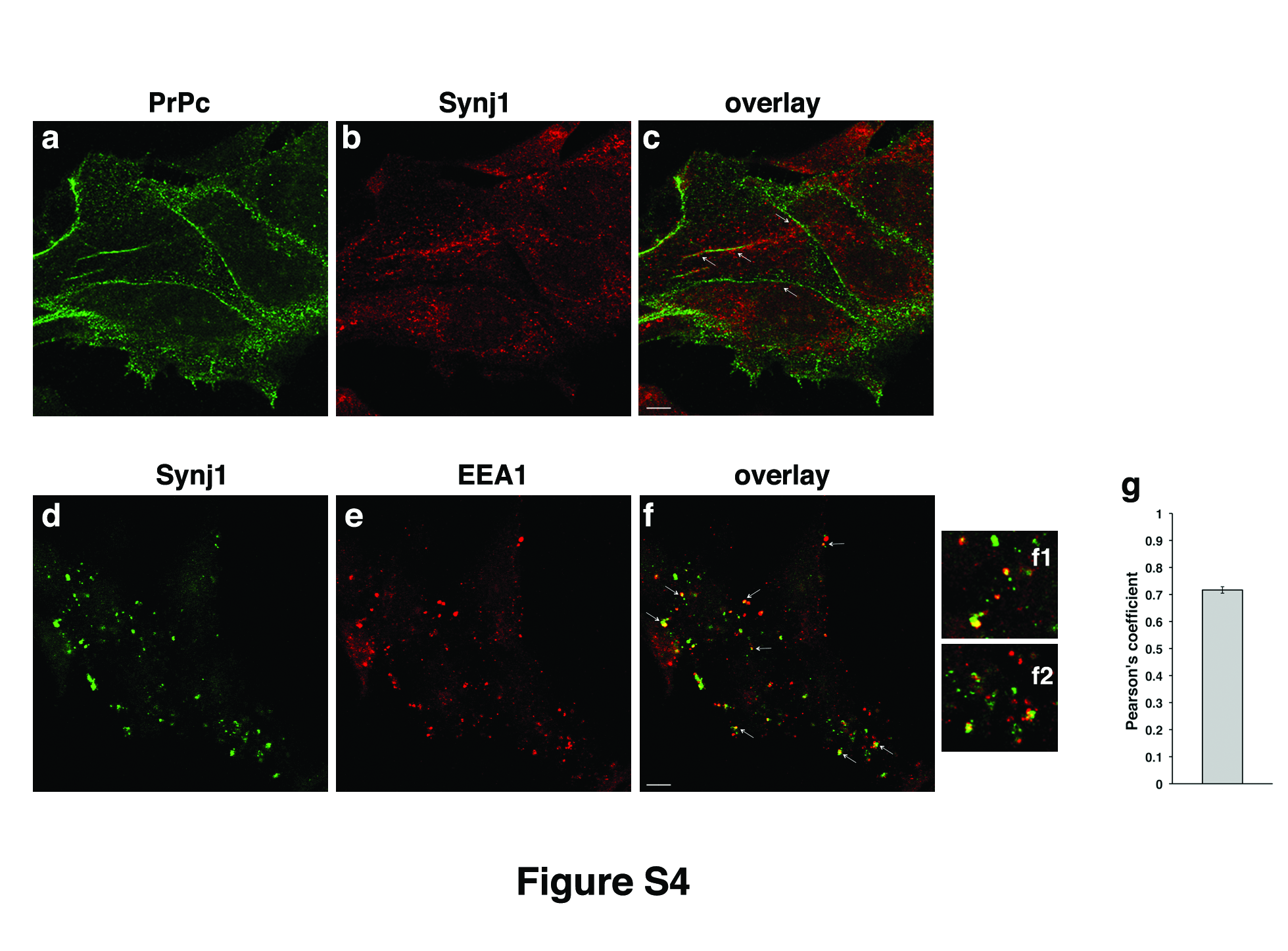

Supplement: Supplementary file 5 — Figure S4 [file 41419_2018_410_MOESM5_ESM.tif]

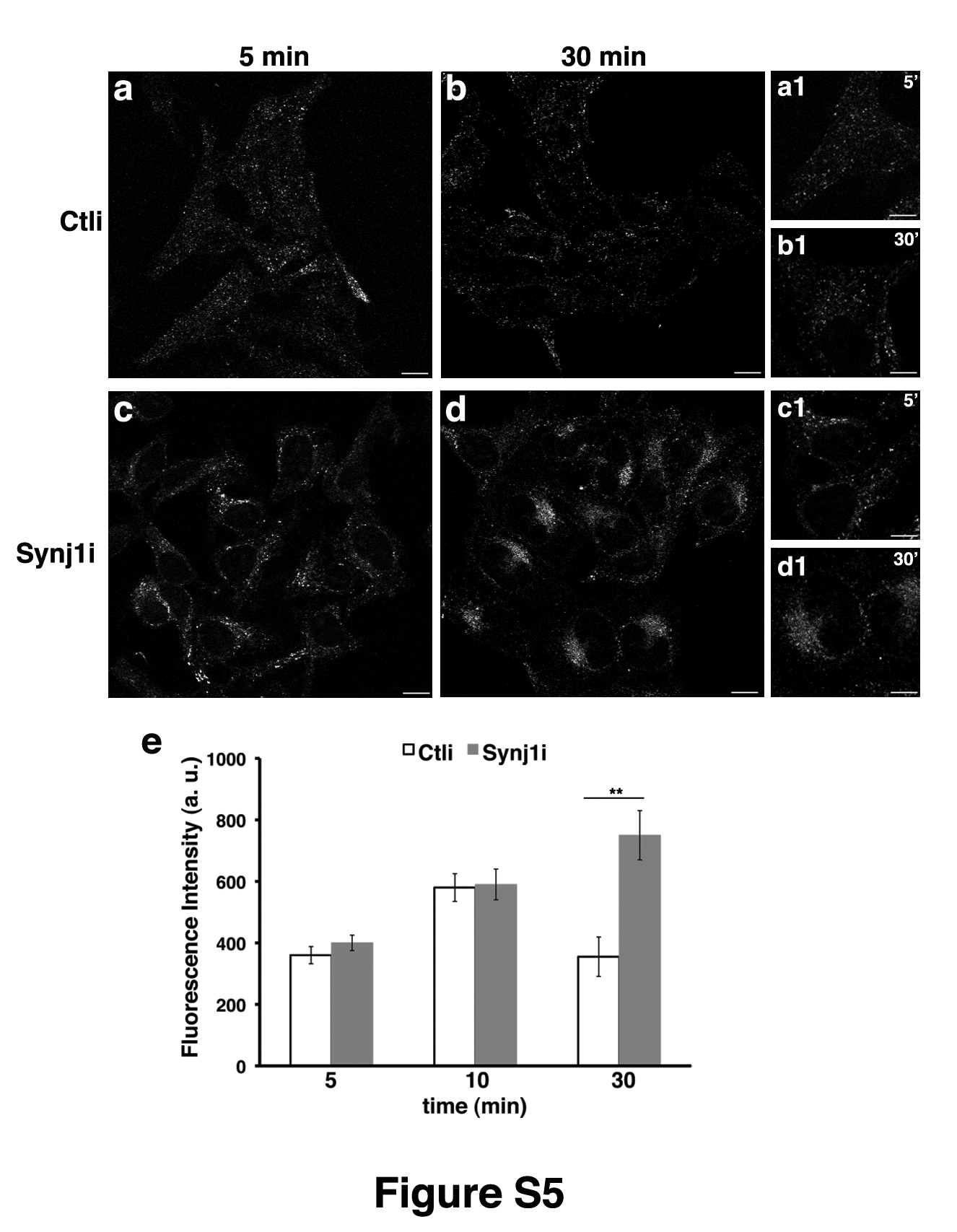

Supplement: Supplementary file 6 — Figure S5 [file 41419_2018_410_MOESM6_ESM.tif]

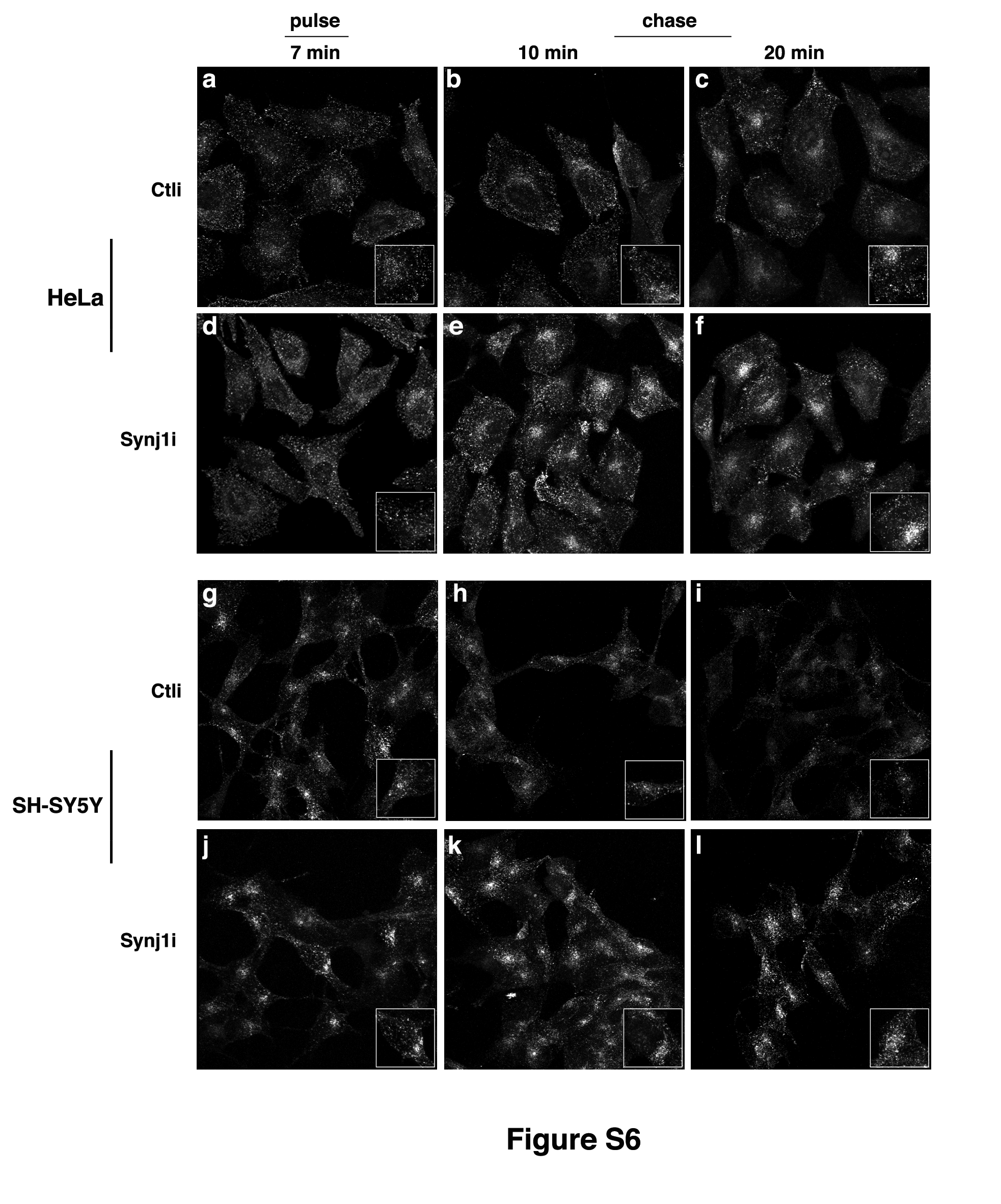

Supplement: Supplementary file 7 — Figure S6 [file 41419_2018_410_MOESM7_ESM.tif]

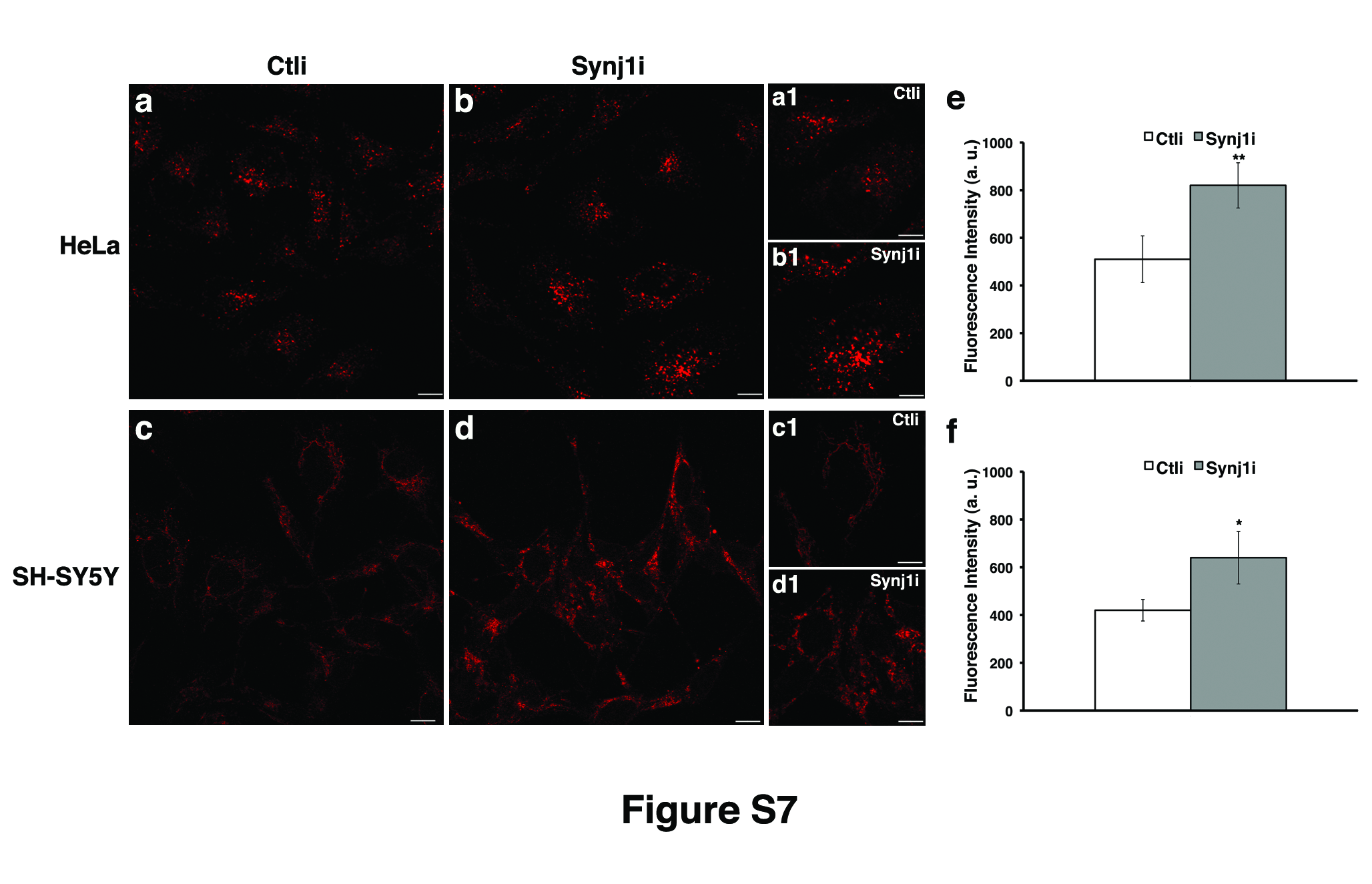

Supplement: Supplementary file 8 — Figure S7 [file 41419_2018_410_MOESM8_ESM.tif]
